# 3D spheroid models of paediatric SHH medulloblastoma mimic tumour biology, drug response and metastatic dissemination

**DOI:** 10.1038/s41598-021-83809-6

**Published:** 2021-02-19

**Authors:** Sophie J. Roper, Franziska Linke, Paul J. Scotting, Beth Coyle

**Affiliations:** 1 Children’s Brain Tumour Research Centre, Biodiscovery Institute, University of Nottingham, Nottingham, UK; 2 School of Life Sciences, University of Nottingham, Nottingham, UK

**Keywords:** Cancer models, CNS cancer

## Abstract

Studying medulloblastoma, the most common malignant paediatric brain tumour, requires simple yet realistic in vitro models. In this study, we optimised a robust, reliable, three-dimensional (3D) culture method for medulloblastoma able to recapitulate the spatial conformation, cell–cell and cell–matrix interactions that exist in vivo and in patient tumours. We show that, when grown under the same stem cell enriching conditions, SHH subgroup medulloblastoma cell lines established tight, highly reproducible 3D spheroids that could be maintained for weeks in culture and formed pathophysiological oxygen gradients. 3D spheroid culture also increased resistance to standard-of-care chemotherapeutic drugs compared to 2D monolayer culture. We exemplify how this model can enhance in vitro therapeutic screening approaches through dual-inhibitor studies and continual monitoring of drug response. Next, we investigated the initial stages of metastatic dissemination using brain-specific hyaluronan hydrogel matrices. RNA sequencing revealed downregulation of cell cycle genes and upregulation of cell movement genes and key fibronectin interactions in migrating cells. Analyses of these upregulated genes in patients showed that their expression correlated with early relapse and overall poor prognosis. Our 3D spheroid model is a significant improvement over current in vitro techniques, providing the medulloblastoma research community with a well-characterised and functionally relevant culture method.

## Introduction

Medulloblastoma is the most common malignant paediatric brain tumour, accounting for around 10% of cancer-related deaths in children^1^. Although once regarded as a single tumour entity, it is now widely accepted that there are four distinct molecular subgroups of medulloblastoma (WNT, SHH, Group 3, and Group 4), which differ in their patient demographics, metastatic potential, and prognosis^2,3^. All medulloblastoma tumours are categorised as Grade IV, irrespective of their histological or molecular characteristics, attesting to their malignant and aggressive behaviour. In addition, advances in molecular and genetic profiling have shown considerable intertumoral heterogeneity in medulloblastomas and highlighted the need to rethink patient risk stratification^4,5^. It is now proposed that patients are stratified into low, standard, high, and very high risk groups and that treatment regimens should be adjusted accordingly^6^. Current treatment protocols for paediatric medulloblastoma include maximal safe surgical resection with a combination of craniospinal radiotherapy and adjuvant chemotherapy^7^. Unlike in older children, the use of radiotherapy is avoided for infants (patients < 3 years of age) due to the neurocognitive defects associated with radiation-induced damage to the developing brain^1^. In addition, the high levels of toxicity of standard-of-care chemotherapeutics are driving the need to develop and assess alternative drugs for medulloblastoma treatment.

In order to improve our understanding of medulloblastoma tumour biology and behaviour, it is important to adopt a suitable culture system. The majority of in vitro experiments for medulloblastoma and other tumour types have been performed in two-dimensional (2D) monolayer culture. Although reproducible and amenable for high-throughput drug screening studies, 2D culture fails to recapitulate the multi-dimensional growth, physiological gradients, and cell–cell interactions that exist in animal models and in patient tumours and can therefore over/under-estimate the therapeutic efficacy of compounds. Over recent years, there have been vast improvements in in vitro technologies, allowing researchers to grow cancer cells in three-dimensional (3D) culture. 3D culture aims to mimic the characteristics observed in patient tumours to provide a more predictive model of tumour biology, thus filling the gap between standard 2D monolayer culture and animal models.

There are numerous 3D in vitro modelling systems available and each of these methods have their own uses as well as advantages and disadvantages, as summarised in Supplementary Table S1. The 3D spheroid-based model, in which cell aggregates are grown in suspension, was pioneered by Sutherland et al. in the early 1970s^8^. Since then, the technique has been widely utilised in disease modelling, drug evaluation, and studying cell migration and invasion^9–13^. Not only is this method widely accessible due to its cost effectiveness, but it is also easily reproducible and long-term culture is possible. While many cancer cell lines have been optimised for 3D spheroid growth^10^, there have been limited studies utilising medulloblastoma cell lines in this model^14–19^. In this study, we therefore aimed to respond to the need for an optimised and well-characterised 3D spheroid model of medulloblastoma. We demonstrate how established cell lines can be utilised in this model to study growth kinetics, identify differences in drug response that are only apparent in 3D culture, and model the initial stages of metastatic dissemination.

## Results

### Optimisation and characterisation of a 3D spheroid model of SHH medulloblastoma

**Figure 1:**
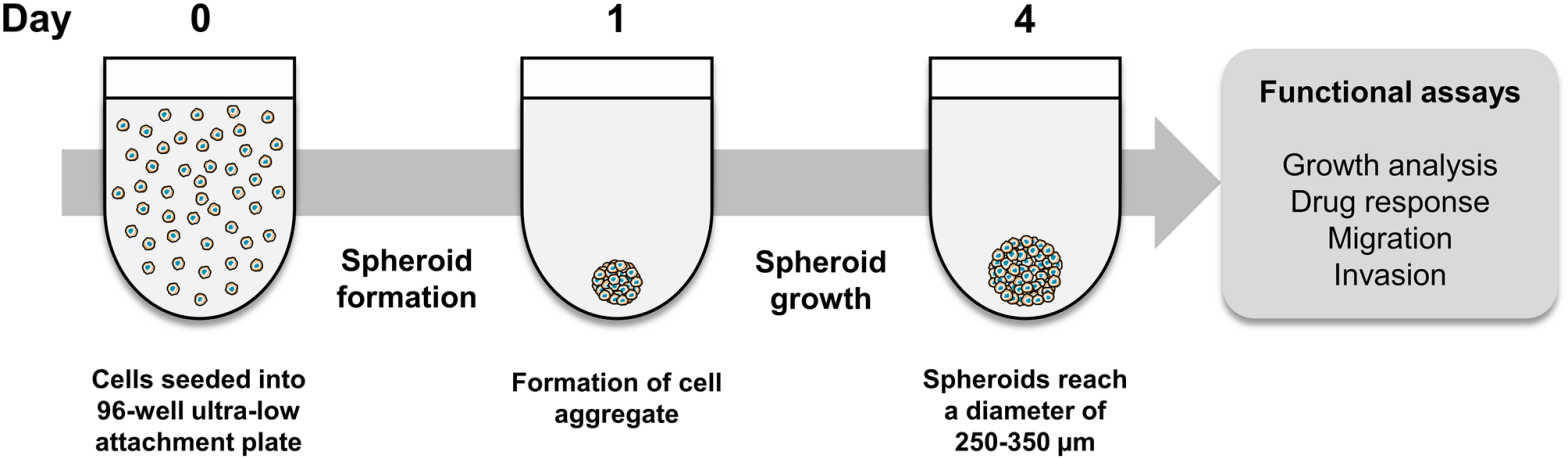
Schematic diagram describing the establishment of the 3D spheroid model and its applications. On day 0, cells are harvested and seeded into 96-well ultra-low attachment, round-bottom plates at an optimal cell density to achieve 3D spheroids with a diameter of 250–350 µm by day 4. 3D spheroids can then be used in numerous functional assays, including growth and drug response analysis, as well as migration and invasion assays using matrices.

The first stage in the establishment of a 3D spheroid model of medulloblastoma was to determine which generation method to adopt. The most common spheroid generation techniques are summarised in Supplementary Table S2. We chose a spontaneous formation method using non-adherent ultra-low attachment (ULA) round bottom plates. These plates have a covalently-bound hydrogel layer to effectively inhibit cellular attachment, thus promoting the formation of a single, highly-reproducible 3D spheroid in each well. This method was previously shown to be superior to traditional agar-coated plates for the spontaneous formation of 3D spheroids^10^. A schematic diagram summarising the 3D spheroid model of medulloblastoma is shown in Fig. 1. On day 0, cell lines grown in their standard culture conditions were harvested to generate a single-cell suspension and were then seeded into 96-well ULA plates. Cells typically formed an aggregate within 24 h and cell densities were optimised to produce 3D spheroids with a diameter of 250–350 µm by day 4, a size considered optimal for the establishment of pathophysiological oxygen gradients^13^. Having reached their optimal size on day 4, 3D spheroids were then suitable for use in numerous functional assays.

**Figure 2:**
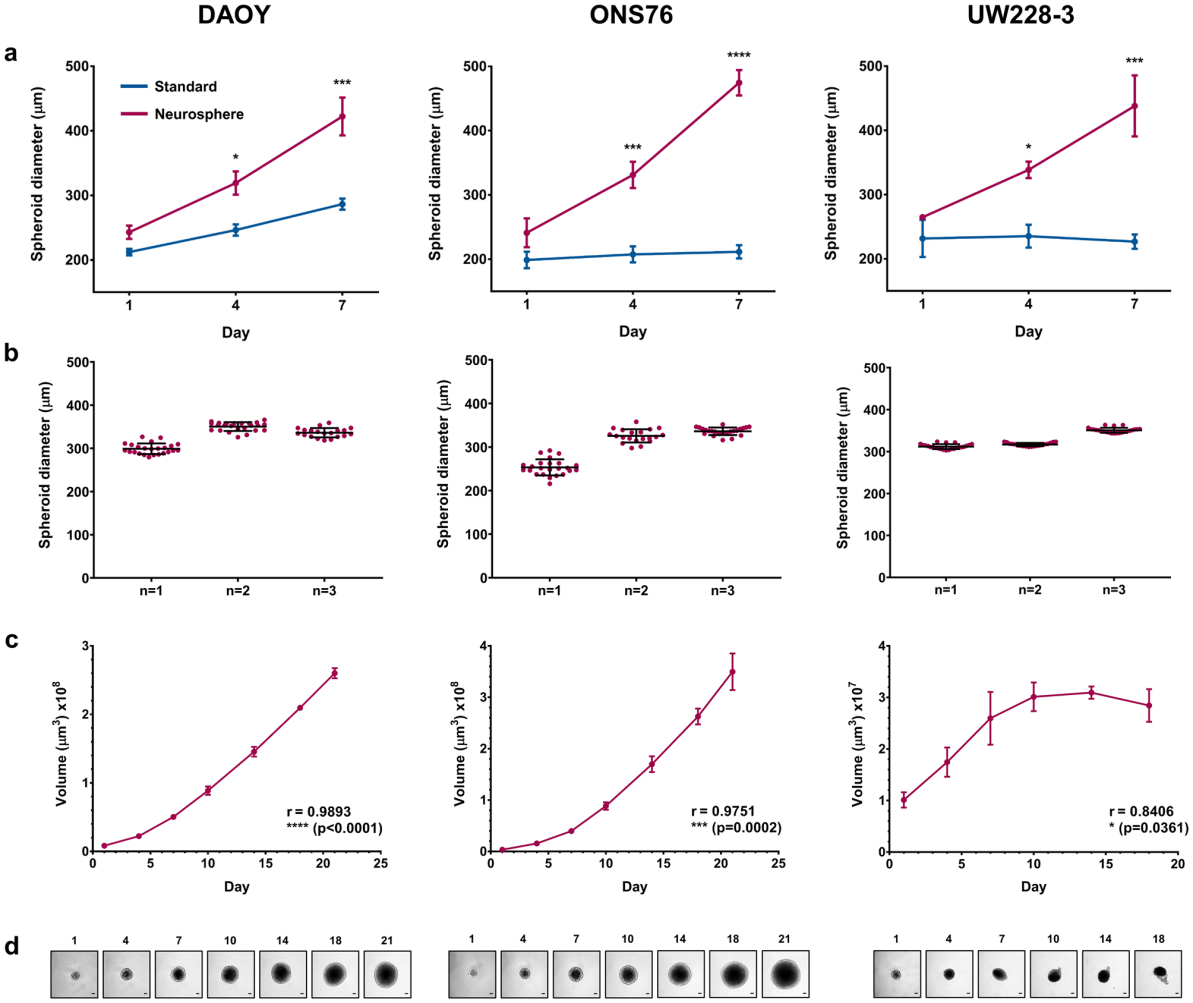
Growth characterisation of SHH medulloblastoma 3D spheroids. (**a**) Growth of SHH medulloblastoma 3D spheroids seeded at their optimal cell density in either neurosphere (red) or standard (blue) medium was assessed over a seven-day period. Spheroid diameter (µm) was significantly enhanced in neurosphere medium for all cell lines. Error bars represent the mean ± SEM of n = 3 experiments each containing 3 replicates. Significance was calculated using two-way ANOVA analyses with Sidak’s multiple comparisons post-hoc test (*p ≤ 0.05, ***p ≤ 0.001, ****p ≤ 0.0001). (**b**) Scatter plots displaying little variation in 3D spheroid diameter on day 4 over three independent experimental repeats (n = 1, 2, and 3) for the SHH medulloblastoma cell lines DAOY, ONS76, and UW228-3. Error bars represent the mean ± SD across plates containing 20–24 3D spheroids. (**c**) Growth analysis of 3D spheroids up to 21 days in culture. Relationship between 3D spheroid volume (µm^3^) and time was assessed using Pearson’s correlation coefficient (r). Error bars represent the mean ± SEM (n = 4) containing 6 replicates at each time point. (**d**) Representative images of 3D spheroids at each time point are shown (scale bar 100 µm).

We tested three established SHH medulloblastoma cell lines (DAOY, ONS76, and UW228-3) for use in the 3D spheroid model. For a cell line to be considered optimised, the 3D spheroids needed to exhibit a suitable morphology (a tight spheroid or compact aggregate, as described previously by Ivascu and Kubbies^20^) as well as maintain continuous growth for at least seven days in culture (the minimum length of time to perform downstream experiments). To determine the optimal 3D spheroid growth conditions for the SHH medulloblastoma cell lines, cells were seeded at increasing densities and the average spheroid diameter on day 4 was measured (Supplementary Fig. S1). We demonstrated that the SHH medulloblastoma cell lines formed 3D spheroids in standard culture media; however, they failed to grow significantly over a seven day period. This is in contrast to cell lines of other tumour types which have been shown to successfully form 3D spheroids and maintain growth in standard serum-containing culture media^10^. However, when seeded in serum-free neurosphere media, containing supplements and growth factors routinely used in the culture and maintenance of neural stem cells, SHH medulloblastoma cell lines formed larger 3D spheroids which displayed continual growth over the same time period (Fig. 2a). A further six established medulloblastoma cell lines were tested for their suitability in the 3D spheroid model as shown in Supplementary Table S3. Some cell lines were unsuitable for this method of culture, despite modifications to promote the formation of a tighter 3D structure (Supplementary Fig. S2); however, five cell lines, including two group 3 lines (DAOY, ONS76, UW228-3, HD-MB03, and D458) were fully optimised.

We next aimed to characterise the reproducibility and growth of the SHH medulloblastoma cell lines in 3D spheroid culture. Coefficient of variation (CV) analysis was used to determine the uniformity of spheroids both within (intraplate CV) and between (interplate CV) experimental repeats. This unit of measurement has been routinely used to assess the reproducibility of spheroids and Sittampalam et al. suggested that less than 20% variation in spheroid size was acceptable^21^. There was minimal variation in spheroid diameter both within and across experimental repeats for all three cell lines (Fig. 2b). Both the intraplate (DAOY: 2.91–4.13%, ONS76: 2.59–7.31%, UW228-3: 1.28–1.88%) and interplate (DAOY: 7.60%, ONS76: 13.33%, UW228-3: 5.67%) CV values calculated were all within the acceptable range (Supplementary Table S4).

**Figure 3:**
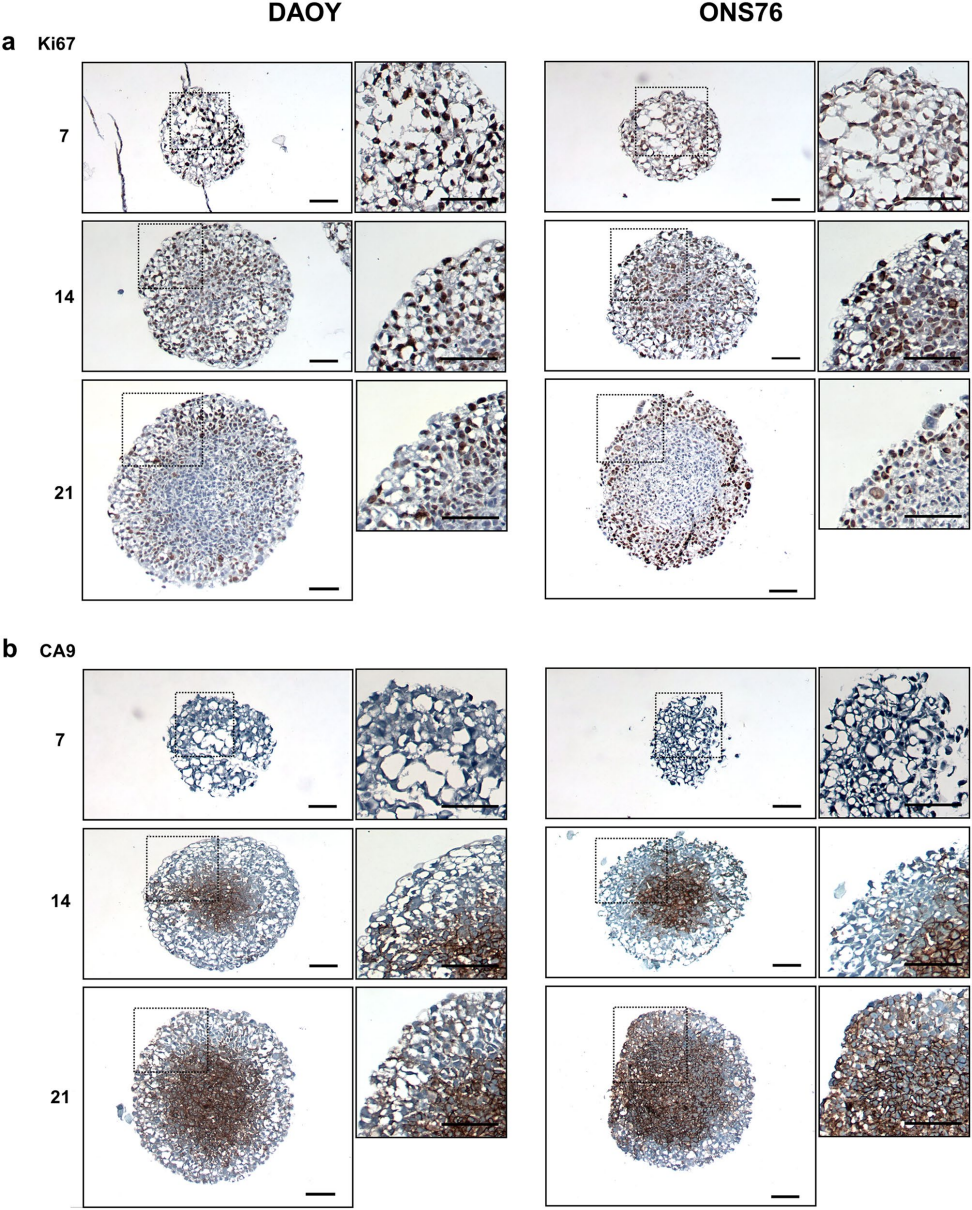
Immunohistochemical staining of SHH medulloblastoma 3D spheroids for markers of proliferation and hypoxia. DAOY and ONS76 3D spheroids were harvested on days 7, 14, and 21, sectioned, and stained with markers of (**a**) proliferation (Ki67) and (**b**) hypoxia (CA9) (scale bar 100 µm). For both cell lines, Ki67 expression was visible throughout the 3D spheroid on days 7 and 14, but expression was restricted to the periphery by day 21. CA9 core expression was present by day 14 and became more extensive by day 21.

One of the main advantages of 3D in vitro models is their ability to remain in culture for a longer period of time compared to standard 2D monolayer cultures which require passaging every 3–4 days to maintain viability. We showed that DAOY and ONS76 3D spheroids continually grew for at least three weeks in culture and UW228-3 3D spheroids maintained growth for around 10 days (Fig. 2c,d). The 3D spheroid growth patterns are similar to those observed when these cell lines are implanted into mouse models. Our 3D model is therefore recapitulating similar growth patterns seen in vivo^22^. In addition, the DAOY and ONS76 3D spheroids developed physiological oxygen gradients within the structure over this time period. Immunohistochemical staining of 3D spheroid sections showed Ki67 expression, a marker of proliferation, throughout the spheroids on days 7 and 14; however, there was an expression gradient by day 21 where Ki67 was restricted to the spheroid periphery (Fig. 3a). Expression of CA9, a hypoxia marker, was evident after two weeks of spheroid culture and was more extensive by day 21, indicating the presence of a hypoxic core at these later time points (Fig. 3b).

As a final characterisation step, we analysed the expression of Hedgehog GLI pathway markers in SHH medulloblastoma 3D spheroids. mRNA sequencing analysis showed the expression of numerous Hedgehog GLI pathway genes, providing evidence that the cell lines used in this study could be categorised as SHH subgroup (Supplementary Table S5).

In this section, we have determined the optimal growth conditions, including culture medium and seeding densities, of SHH medulloblastoma cell lines in the 3D spheroid model. We have also demonstrated that these 3D spheroids retain markers of the Hedgehog GLI pathway are highly reproducible, suitable for longer term culture, and display physiological gradients consistent with spheroid size over time. Together, these characteristics make SHH medulloblastoma 3D spheroids an ideal model for downstream functional experiments.

### SHH medulloblastoma 3D spheroids are improved models of drug response

Pre-clinical testing of potential therapeutic agents for cancer treatment typically involves in vitro screening of hundreds of drugs, with the most successful taken forward for in vivo testing. However, a significant number of drugs which pass in vitro tests later fail in animal models. Additionally, more than 90% of potential anticancer drugs that enter human trials fail to translate to the clinic^23^. The development of more appropriate cellular models that recapitulate tumour biology could positively influence pre-clinical testing by providing a more accurate prediction of drug response at the earliest stage. The optimised growth conditions and identical starting sizes make the SHH medulloblastoma 3D spheroids ideal models for drug screening studies to allow direct comparisons across cell lines and drug combinations^9^. Assessing drug response in 3D spheroids is possible using both size analysis and commercially-available viability assays as measures of efficacy. We showed that both continual size analysis and the endpoint CellTiter-Glo 3D cell viability assay (Promega) are comparable measures of drug response (Supplementary Table S6). The decision regarding which method to adopt should therefore be based on the individual requirements of each experiment and whether intact 3D spheroids are required for further analysis after treatment.

**Figure 4:**
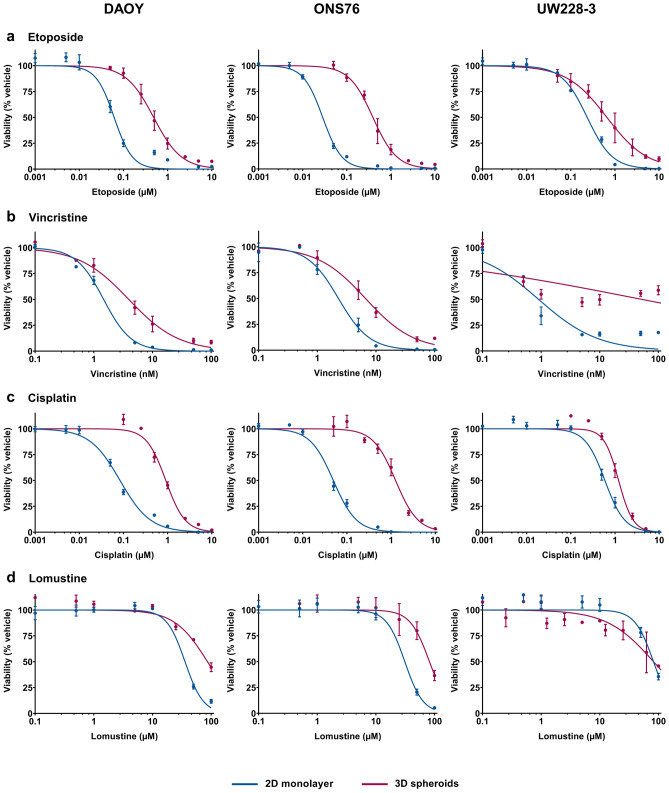
Response to standard-of-care chemotherapeutics in 2D monolayer versus 3D spheroid culture. DAOY, ONS76, and UW228-3 medulloblastoma cell lines were treated with increasing concentrations of (**a**) etoposide, (**b**) vincristine, (**c**) cisplatin, and (**d**) lomustine in both 2D monolayer (blue) and 3D spheroid (red) culture for 72 h. The CellTiter-Glo 3D cell viability assay was performed to compare responses and viability was calculated as a percentage of the vehicle-treated controls. Dose response curves were generated using non-linear regression analyses and error bars represent the mean ± SEM of n = 3 experiments each containing 3 replicates.

As standard 2D culture fails to recapitulate the multi-dimensional growth of tumours, we hypothesised that drug efficacy would differ between cells grown as 2D monolayers and 3D spheroids. We therefore tested the response of the SHH medulloblastoma cell lines in both culture conditions to four chemotherapeutic agents. Drugs (etoposide, vincristine, cisplatin, and lomustine) were selected on the basis that they are all currently used standard-of-care treatments for paediatric medulloblastoma and they differ in their mechanisms of action. We showed that there was a significant difference (p ≤ 0.0001) in drug response with 3D spheroids being more resistant to treatment compared to 2D monolayer culture in almost all cell lines and drugs tested (Fig. 4; Supplementary Table S7).

**Figure 5:**
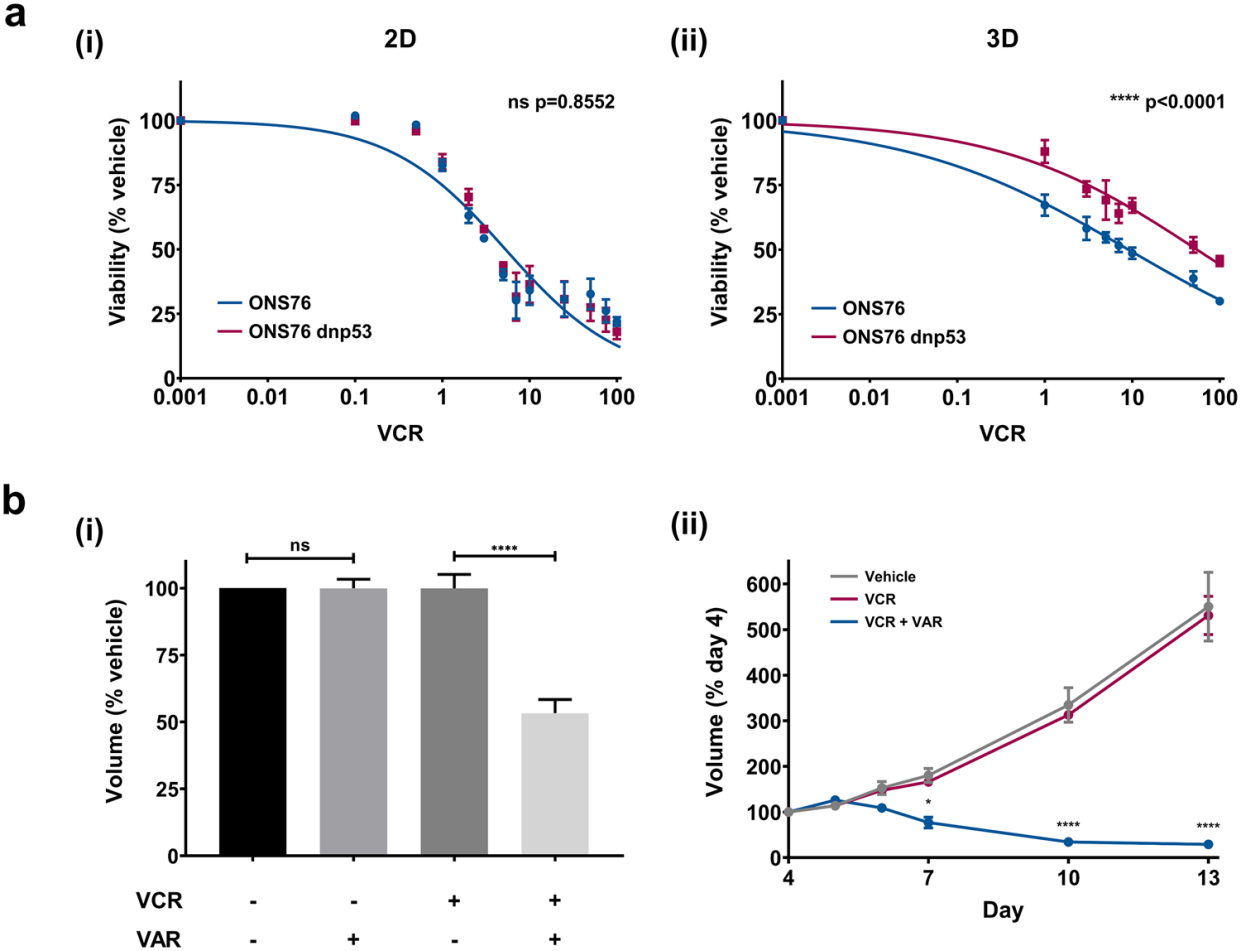
TP53 and ABCB1 modulated drug response can be modelled in 3D spheroids. (**a**) ONS76 (blue) and ONS76 dnp53 (red) cell lines were treated with increasing concentrations of vincristine in both 2D monolayer (i) and 3D spheroid (ii) culture. The CellTiter-Glo 3D cell viability assay was performed to compare responses and viability was calculated as a percentage of the vehicle-treated controls. There was a significant difference in drug response between the cell lines in 3D, but not 2D, culture. Dose response curves were generated using non-linear regression analyses and error bars represent the mean ± SEM of n = 3 experiments each containing 3 replicates. (**b**) (i) ONS76 3D spheroids were treated with vincristine (VCR; 1 nM) in combination with vardenafil (VAR; 10 µM) for 72 h. Images were taken immediately after treatment to measure changes in spheroid volume relative to the vehicle-treated control. There was no difference in volume after vardenafil-only treatment. Vincristine efficacy could be potentiated when treated in combination with vardenafil. Error bars represent the mean ± SEM of n ≥ 3 experiments each containing 3 replicates. Significance was calculated using one-way ANOVA analyses with Sidak’s multiple comparisons post-hoc test (ns = not significant, ****p ≤ 0.0001). (ii) Continual monitoring of the effects of combined vincristine and vardenafil treatment (blue) showed a significant continual reduction in spheroid volume following treatment compared to vincristine alone (red). Significance differences between single and combination treatment were calculated using repeated measures two-way ANOVA analyses with Tukey’s multiple comparisons post-hoc test (*p ≤ 0.05, ***p ≤ 0.01, ****p ≤ 0.0001).

We next aimed to confirm that the observed differences in drug response between 2D monolayers and 3D spheroids were not simply an artefact of 3D culture, but that 3D spheroids are more accurate and reliable models of drug response. To do this, we chose to focus on one of the most significant genetic prognostic indicators for paediatric SHH medulloblastoma: *TP53* mutational status. Compared to *TP53*-wildtype SHH medulloblastoma patients, those with *TP53*-mutant SHH tumours have a profoundly worse outcome^24^. *TP53*-mutated SHH tumours are stratified as very-high risk and account for the majority of treatment failures within the SHH subgroup^25^. To explore whether differences in drug response between *TP53*-wildtype and *TP53*-mutated SHH medulloblastoma cells could be recapitulated in vitro, we utilised ONS76 cells (*TP53*-wildtype) and a genetically modified ONS76 cell line harbouring a dominant negative p53 mutation (*TP53*-mutant; ONS76 dnp53)^26^. We compared vincristine response in both cell types in 2D monolayer and 3D spheroid culture (Fig. 5a). In 2D monolayer culture, there was no difference in response between ONS76 wildtype and dnp53 cells (p = 0.8552). However, there was a significant difference in response in 3D spheroid culture, with ONS76 cells being more sensitive to vincristine treatment than ONS76 dnp53 cells (p < 0.0001). Together, these results indicate that 3D spheroid culture is able to identify differences in drug response that are not evident in 2D monolayer culture.

So far, we have demonstrated the testing of single compounds in the 3D spheroid model; however, it is possible to test inhibitor combinations. To demonstrate this approach, we aimed to potentiate the effects of vincristine through combined treatment with vardenafil, an inhibitor of the multi-drug transporter ABCB1 (for which vincristine is a substrate^27^). ONS76 3D spheroids were treated with vincristine (1 nM; the measurable concentration in patient CSF^28^) and/or vardenafil (10 µM; the effective concentration as shown by Othman et al.^29^) for 72 h, after which 3D spheroid volume was measured. There was no difference in volume after vardenafil-only treatment in comparison to the vehicle-treated control, therefore showing that vardenafil itself does not affect spheroid viability. However, the effects of vincristine were significantly potentiated when treated in combination with vardenafil compared to vincristine treatment alone (p ≤ 0.0001; Fig. 5b(i)). Continual monitoring of the effects of vincristine versus combined vincristine and vardenafil treatment showed enhanced potentiation of dual-inhibitor therapy over a longer period of time (Fig. 5b(ii)). The same response was also observed for DAOY 3D spheroids (Supplementary Fig. S3). These results not only demonstrate how the 3D spheroid model can be used to monitor drug combinations over time but are also of substantial clinical importance as they highlight the improved efficacy of low dose vincristine when treated in combination with vardenafil.

We have shown that drug response differs in 3D spheroids compared to traditional 2D monolayer culture. We confirmed that this model can identify differences in drug response that would be missed in standard 2D culture studies and highlighted the benefits of monitoring drug response over a longer time period. SHH medulloblastoma 3D spheroids are therefore robust, reliable and improved models for therapeutic screening approaches.

### Genes upregulated in our 3D spheroid migration model are associated with fibronectin, poor prognosis and early relapse in SHH medulloblastoma patients

The predominant mode of medulloblastoma metastasis is leptomeningeal dissemination^30^. SHH medulloblastomas typically spread across the leptomeningeal surface; however, the mechanisms by which this occurs is poorly understood. The initial stages of tumour spread generally involves the alteration of the cell’s cytoskeleton, increased cell motility, and interaction with the surrounding extracellular matrix (ECM). It is possible to study these cell–cell and cell–ECM interactions in vitro using 3D spheroids by modelling their migration across different matrices in order to gain insights into the processes involved in tumour spread^13^.

Numerous commercially-available matrices such as Matrigel and Basement Membrane Extract have been used for modelling cancer cell migration in many tumour types. However, these matrices are prone to batch variability and their full composition is confidential. Instead, the use of a simple, one-component, modifiable matrix would be more suitable for establishing a robust technique for modelling tumour cell dissemination. In this study, we chose to utilise a hyaluronan (hyaluronic acid; HA) hydrogel for modelling 3D spheroid migration. HA is a major constituent of the brain ECM^31,32^ and therefore serves as a tissue-relevant matrix, with the additional advantage that this matrix can be modified to mimic the brain’s stiffness.

**Figure 6:**
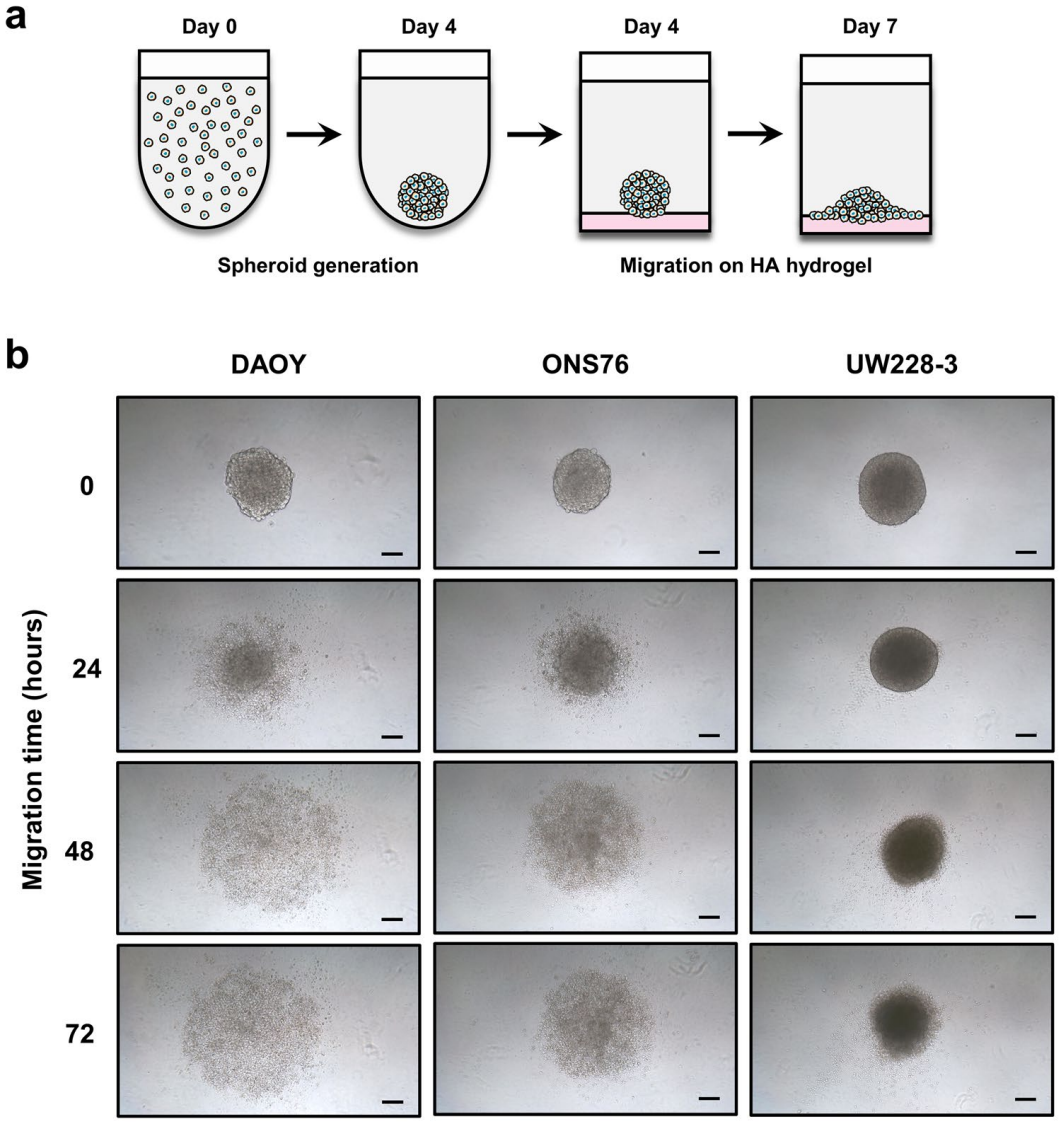
Modelling tumour cell dissemination on hyaluronan hydrogels using 3D spheroids. (**a**) Schematic diagram describing the hyaluronan (HA) spheroid migration model. Cells were seeded on day 0 and the resulting 3D spheroids were transferred to 96-well flat-bottom plates coated with HA hydrogel. Cell migration from the 3D spheroid across the HA hydrogel surface is then visualised over time. (**b**) Representative images of DAOY, ONS76, and UW228-3 3D spheroids migrating across HA hydrogels over a 72 h period. Images of the same 3D spheroid at different time points are shown (scale bar 100 µm) and are representative of n = 3 experiments each containing 3 replicates.

A schematic diagram describing the tumour cell dissemination model of 3D spheroid migration across HA hydrogels is shown in Fig. 6a. 3D spheroids were generated as described and on day 4, were transferred to HA hydrogel-coated flat-bottomed 96-well plates. Cell migration from the 3D spheroid across the matrix was then assessed over a 72 h period. DAOY and ONS76 cells actively migrated from the main spheroid body and across the HA hydrogel within 24 h and continued to migrate after 72 h (Fig. 6b). However, migration was limited for the non-metastatic UW228-3 3D spheroids.

**Figure 7:**
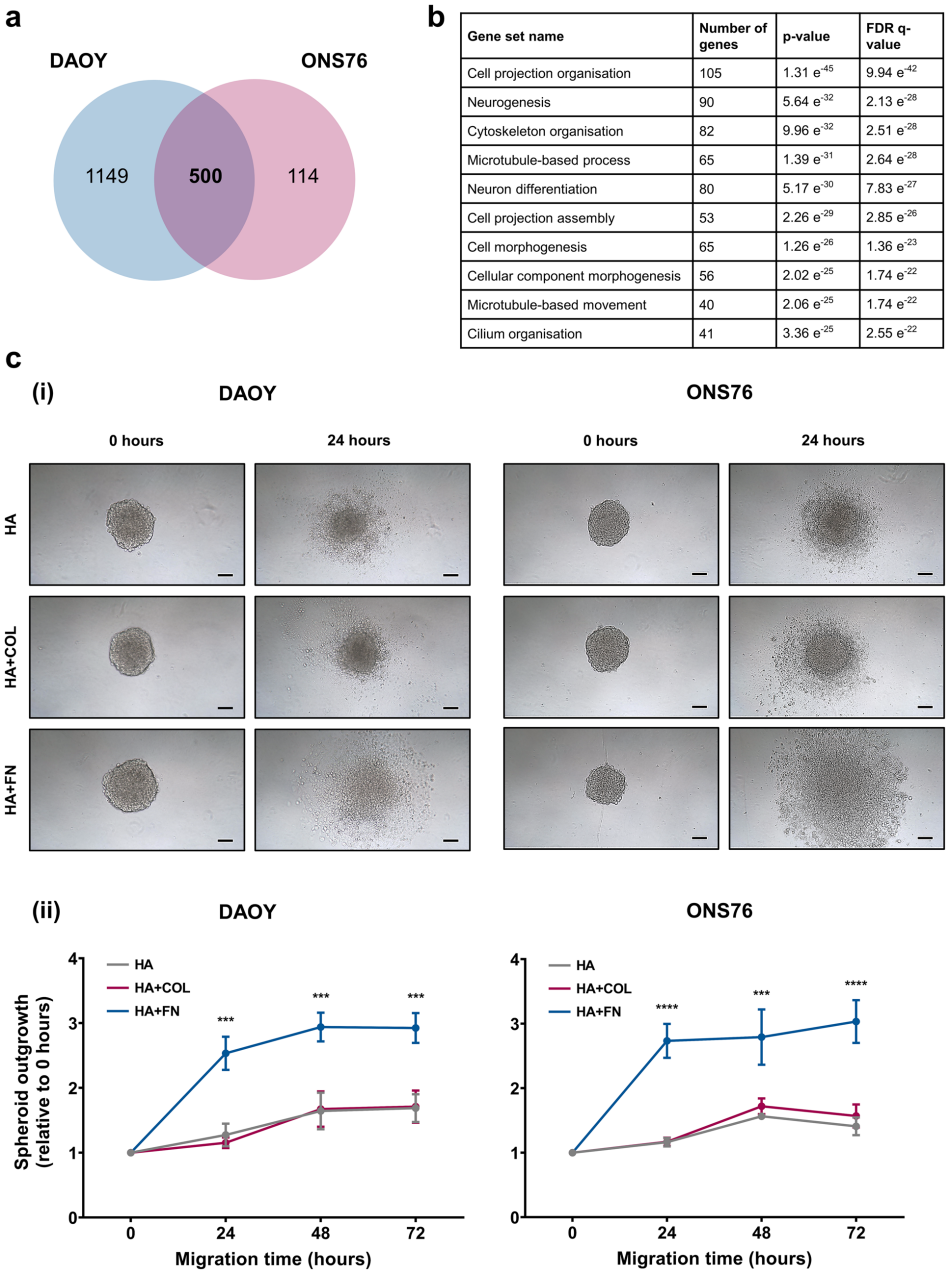
Cell movement genes, including those associated with fibronectin interactions, are upregulated genes in the 3D spheroid migration model. Differential gene expression analysis was conducted to identify upregulated genes in the 3D spheroid migration model. (**a**) 500 upregulated genes (Log2 fold change ≥ 1) were shared between DAOY and ONS76 samples. (**b**) Top 10 Gene Ontology (GO) biological process gene sets significantly associated with these 500 upregulated genes was assessed using Gene Set Enrichment Analysis. Significances were determined using an uncorrected p-value and a q-value (a p-value adjusted using the Benjamini–Hochberg False Discovery Rate approach) to correct for multiple testing. (**c**) Spheroid migration across hyaluronan (HA) hydrogels containing collagen I (COL) or fibronectin (FN) was visualised and quantified. (i) Representative images of DAOY and ONS76 3D spheroids migrating across the surface of hyaluronan hydrogels over a 24 h period. Images of the same 3D spheroid at each time point are shown (scale bar 100 µm) and are representative of n = 3 experiments each containing 3 replicates. (ii) Spheroid outgrowth was calculated by measuring the area covered by migrating cells and normalising to the initial spheroid size at 0 h. Significant differences in relative spheroid outgrowth were calculated using two-way ANOVA analyses with Tukey’s multiple comparisons post-hoc test (***p ≤ 0.01, ****p ≤ 0.0001).

Having established that DAOY and ONS76 3D spheroids migrate across HA hydrogels, we then used next-generation sequencing (NGS) technologies to gain an insight into the global gene expression changes occurring in this 3D spheroid migration model. More details regarding sample preparation and the approaches adopted for NGS are included in the Supplementary Methods S1. Differential gene expression analysis revealed the upregulation of genes associated with cell movement (Fig. 7a,b) and the downregulation of cell cycle processes (Supplementary Fig. S4) in the 3D spheroid migration model compared to spheroids grown in suspension. Lists of the up- and down-regulated genes and associated gene sets identified in this analysis are included in Supplementary File S1. Evaluation of the list of upregulated genes in the 3D spheroid migration model identified numerous genes associated with the ECM component fibronectin (FN) that have been previously linked to increased cancer cell movement. These genes include *NOTCH1*^33^, *PAX6*^34^, and *SOX2*^35^. In order to validate the functional relevance of FN on cell migration, we compared the migratory patterns of DAOY and ONS76 3D spheroids on HA matrices with and without FN. Collagen I (COL) was included as a control ECM component as it has been utilised in previous studies of medulloblastoma migration and invasion patterns^18,36^. The addition of FN to the HA matrix significantly enhanced cell dissemination in both cell lines compared to HA and HA + COL matrices (Fig. 7c, Supplementary Fig. S5), highlighting a potential role for FN in medulloblastoma migration and metastasis.

**Figure 8:**
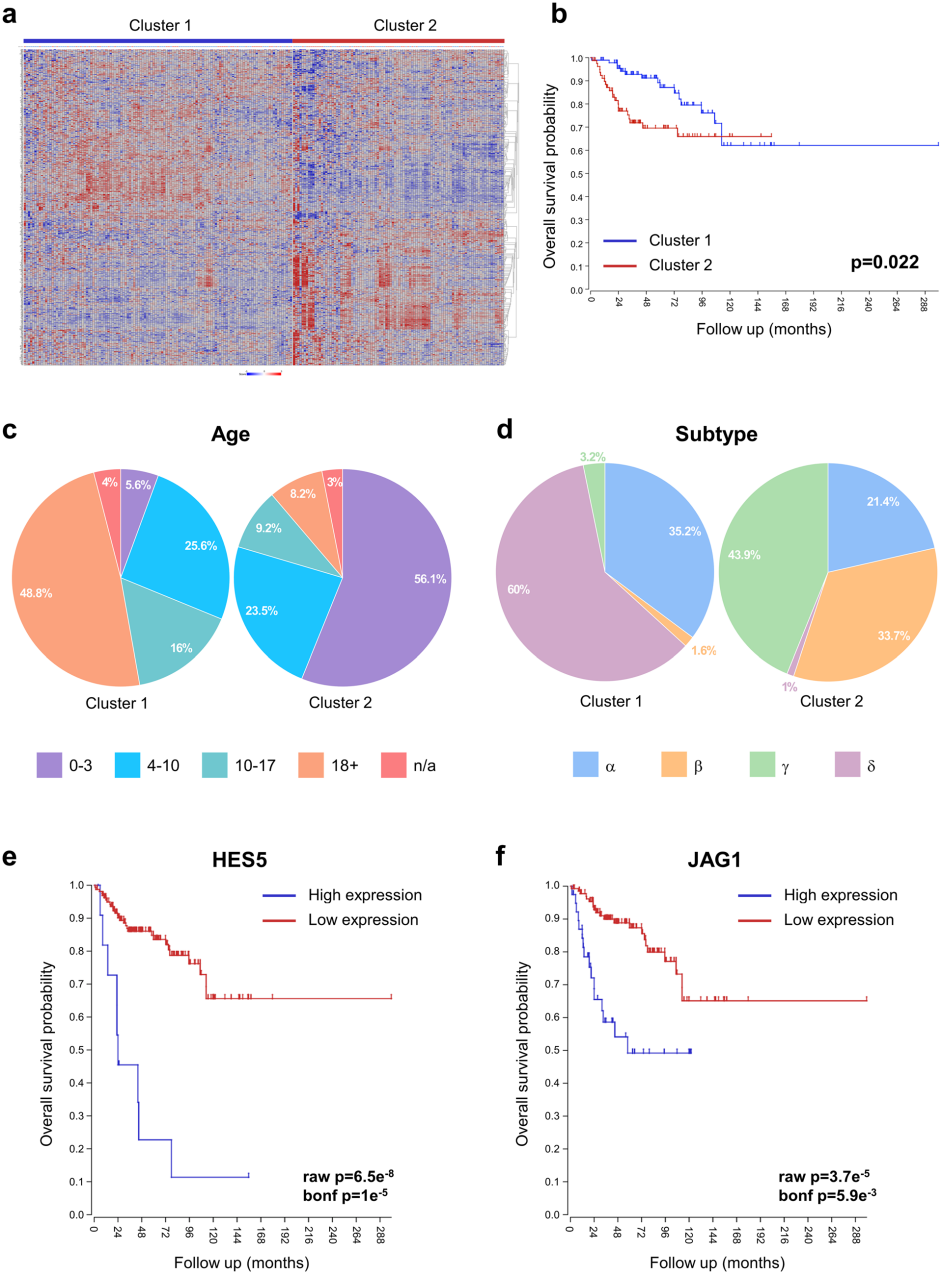
Upregulated genes in the 3D spheroid migration model are associated with poor prognosis and early relapse. The R2 Genomics Analysis and Visualization Platform was used to analyse the Cavalli medulloblastoma dataset. (**a**) Heatmap showing the *k*-means clustering of 223 SHH subgroup samples into two groups (cluster 1: n = 125; cluster 2: n = 98) based on the expression of the 500 upregulated genes in the 3D spheroid migration model. (**b**) Kaplan–Meier overall survival curves for clusters 1 and 2. Pie charts showing the age (**c**) and SHH subtype (**d**) distributions in clusters 1 and 2. Kaplan–Meier overall survival curves for the stem cell markers *HES5* (**e**) and *JAG1* (**f**) in SHH medulloblastoma patients (n = 172).

In order to assess whether the spheroid migration model is representative of patient disease and outcome, we analysed the Cavalli medulloblastoma dataset^4^ using the R2 Genomics Analysis and Visualization Platform. We took the lists of shared up- and down-regulated genes identified in Fig. 7 and performed *k*-means clustering analysis to separate a cohort of SHH medulloblastomas (n = 223) into two clusters (Fig. 8a). For the 500 upregulated genes, the two clusters significantly differed in their overall survival, with a worse outcome associated with patients stratified in cluster 2 (p = 0.022; Fig. 8b). This cluster consisted of predominantly infants (aged 0–3 years) and young children (aged 4–10 years) (Fig. 8c). In addition, almost all SHH-β tumours, the most aggressive subtype associated with metastatic disease and poor outcome, were present in cluster 2 (Fig. 8d). These findings suggest that the upregulated genes we identified in our 3D spheroid migration model can predict poor survival and early relapse in patients. The same analysis when conducted for the genes downregulated in the 3D spheroid migration model, did not associate with any of the above features, as shown in Supplementary Fig. S6.

We also examined the expression of stemness-related genes in the spheroid migration model. Cancer stem cells represent an important subpopulation of cells within a tumour with self-renewal and differentiation capacities, and also play a role in therapy resistance. Supplementary Table S8 details expression data of genes associated with neuronal stem cell population maintenance. Interestingly, DAOY and ONS76 migrating spheroids exhibited upregulation of key stem cell markers including *DLL1*, *HES5*, *JAG1*, *NOTCH1*, *PCM1*, *SOX2*, and *SRRT*. Elevated expression of some of these genes, including the Notch target gene *HES5* and Notch ligand *JAG1*, were correlated with poor survival in SHH patients (Fig. 8e,f).

We conclude that the 3D spheroid migration model of SHH medulloblastoma recapitulates a migratory and stemness phenotype that is reflective of patient disease and prognosis.

## Discussion

3D in vitro culture techniques have been widely described over the past few decades. These cellular models more accurately reflect the spatial conformation of patient tumours and are therefore considered more predictive models of tumour biology than conventional 2D culture methods. In this study we aimed to respond to the need for a robust, reliable and optimised 3D culture technique for SHH medulloblastoma to be utilised in growth assays and drug response studies. In addition, we sought to model tumour spread using a brain-specific HA hydrogel and correlate gene expression patterns with patient outcome.

Our decision on which 3D in vitro culture method to adopt was centred around their suitability for use in the numerous functional assays we wished to perform. Scaffold-based models, in which cells are embedded within a gel or matrix, are ideal for studying cell adhesion and matrix remodelling. In addition, these matrices can be incorporated into transwell-based assays, featuring two chambers separated by a semi-permeable membrane with a chemotactic gradient, to study cell migration and invasion. One of the main advantages of scaffold-based models is the ability to choose organ-specific matrices for the long-term culture of cells. However, this method is expensive, especially when scaled up to higher throughput drug screening approaches. Instead, we chose to adopt a 3D spheroid-based model for our study. This technique comprises of cell aggregates grown in a 3D conformation and can be much more cost-effective than scaffold-based culture techniques. Although there are numerous methods of spheroid generation, we chose to adopt a spontaneous formation method which utilises non-adherent surfaces to allow the formation of 3D spheroids from single cells. One of the main advantages of this method is its accessibility as the only specialised equipment required is an ULA plate, unlike other generation techniques requiring expensive rotary cell culture systems. These ULA plates produce a single 3D spheroid in each well, the size of which can be accurately controlled by adjusting the cell seeding density. The tight regulation of such experimental parameters results in the generation of highly reproducible 3D spheroids ideal for use in numerous experimental workflows, for example long-term growth assays, drug screening, and cell migration/invasion assays.

Having determined the appropriate 3D culture technique, we tested a panel of widely-used medulloblastoma cell lines for use in the 3D spheroid model. Cell line subgroups were recently validated by Linke et al. by RNA sequencing^37^. We firstly adopted the same spheroid generation method and culture conditions described by Vinci et al.^10^ and found that, unlike cell lines of other tumour types, SHH medulloblastoma cell lines form 3D spheroids but fail to grow in serum-containing medium. This gave us the opportunity to test a universal medium which would permit standardised growth conditions across cell lines to allow direct comparisons. We chose to test 3D spheroid formation and growth in “neurosphere medium”, serum-free medium with additional supplements and growth factors, similar in composition to previously published medulloblastoma neurosphere culture media^38,39^. In these conditions we observed a significant difference in 3D spheroid growth, with the three SHH cell lines tested all forming larger spheroids in neurosphere media compared to standard culture media and maintaining continual growth for at least seven days. Importantly, cell seeding densities were optimised to produce 3D spheroids with a diameter of 250–350 µm by day 4, the optimal size for the establishment of pathophysiological oxygen gradients^13^. For all cell lines tested, low cell numbers (ranging from 200 to 1500 cells per well) were required for the formation of suitably sized 3D spheroids. Compared to another study which utilised DAOY cells in a hanging drop 3D spheroid model and required 45,000 cells per droplet^40^, the low cell numbers optimised in our model can easily be scaled up for high-throughput studies. Preliminary results show that SHH medulloblastoma 3D spheroids can be easily cultured in 384-well ULA plates at the same seeding densities optimised in this study (Supplementary Fig. S7).

We observed differences in spheroid formation ability and morphology across medulloblastoma subgroups. SHH cell lines, which grow adherently in standard culture, formed tight, uniform, and highly reproducible spheroids. However, Group 3 and 4 cell lines, which normally grow semi-adherently or in suspension, required additional modifications to tighten their structure or were unsuitable for culture. The observed variation in 3D spheroid morphology across cell lines may therefore reflect the underlying phenotype of each cell type. It was previously shown that more aggressive, invasive cell lines are more likely to form looser 3D structures^41^. Although their morphology may reflect the clinical characteristics and behaviour of such tumours, the use of these 3D spheroids in downstream functional experiments is difficult as loose aggregates typically fall apart when under manipulation. Cells which grow predominantly adherently in standard culture seem more likely to form suitable spheroids. The adhesive characteristics of these cell lines in standard culture may promote stronger cell–cell interactions in a 3D environment which leads to the formation of a tighter spheroid structure. This observation should be tested across other cell lines in order to conclude whether standard cell culture phenotype is a true indicator of expected spheroid morphology. Although some of the cell lines tested did not form tight 3D spheroids, additional supplementation to the culture medium may promote the formation of a more compact aggregate^42,43^.

We demonstrated that SHH medulloblastoma cell lines formed highly reproducible 3D spheroids, making them amenable for drug screening assays^9^. We showed that there was a significant difference in drug response with 3D spheroids being more resistant to treatment compared to 2D monolayer culture in almost all cell lines and drugs tested. This indicated that these shifts in response were unlikely to be drug or cell line specific, but more likely due to the differences in the cellular structures and architecture of the 2D and 3D cultures. Unlike 2D monolayer culture, where cells are exposed to equal levels of nutrients, oxygen, and waste products, the spatial conformation of 3D spheroids generates pathophysiological gradients^44^. An oxygen gradient is established during spheroid growth, with the oxygen availability decreasing with increasing spheroid depth^45^. A low oxygen environment can reduce the cytotoxic effects of some drugs, e.g. cisplatin, and may account for the shift in response in 3D spheroid culture^46^. Oxygen depletion in the spheroid core can cause a shift to anaerobic metabolism, resulting in the accumulation of the by-product lactate within the 3D spheroid^46^. In solid tumours and spheroids, the lactate production results in an acidic environment. For weak basic drugs including vincristine, this results in protonation (the addition of a proton making the drug positively-charged)^47^ and a reduction in uptake as charged drugs are not as effective in bypassing the cellular membrane. Swietach et al. demonstrated this effect in HCT-116 colon cancer spheroids and showed that the uptake of doxorubicin decreased in acidic regions of the spheroid^48^. This pH gradient within the 3D spheroid could have contributed to the shift in drug response in the SHH medulloblastoma cell lines tested. An alternative mechanism of drug resistance in 3D spheroid culture is through cell dormancy, where the lack of oxygen and nutrients and a low pH environment induces quiescence or senescence. DNA damaging agents, for example cisplatin, carboplatin, and doxorubicin, exert their antitumoral effects in actively growing cells^49^. Therefore, the non-proliferative state of dormant cells within the spheroid structure may account for shift in response. In addition, analysis of stemness markers in our RNAseq dataset showed that our 3D spheroid models express *SOX2*, which has been linked to quiescence and therapy resistance in SHH medulloblastoma^50^ and could therefore account for some of the drug resistance observed. However, it would be important to distinguish between drug resistance and a lack of drug penetration in future studies. This could be achieved by fluorescently-labelling compounds and visualising their penetration through the spheroid structure. This technique has been successfully used to study drug and nanoparticle penetration^51,52^ and our 3D spheroid model would be ideally suited for this application. There is also the potential to adopt higher throughput techniques for drug screening, for example the use of automated imaging systems and the use of 384-well ULA plates (Supplementary Fig. S5).

The inclusion of a genetically modified *TP53*-mutated cell line in our study enabled us to identify a difference in 3D drug response that was not evident in 2D monolayer culture. Having the ability to detect variability in drug response could positively influence drug screening studies, highlighting particular subgroups of patients who may respond differently to therapy and the adjustment of treatment regimens accordingly. We also demonstrated how longer term monitoring can reveal differences in drug response that could be missed in endpoint assays. This was particularly apparent when potentiating the effect of vincristine with the ABCB1 inhibitor vardenafil. In future studies, longer term drug studies could be used to continually drug treat remaining cells, generating a treatment-resistant 3D spheroid population.

Our final aim of this study was to model SHH medulloblastoma metastatic dissemination using 3D spheroids. Although matrices such as Matrigel or BME are widely used, the exact concentration of its components is unknown and the resulting batch-to-batch variability is a significant drawback of the technique. In addition, as these matrices are derived from EHS murine chondrosarcoma tumours, they are not physiologically relevant for medulloblastoma tumours. Hydrogels, water-swollen networks of polymers, are alternative matrices for 3D culture in which cells can be placed on top of or encapsulated within the gels, making them amenable for spheroid migration and invasion modelling. There has been limited research on the use of hydrogels for the culture of medulloblastoma cell lines. Three publications have described the use of the MAX8 β-hairpin self-assembling peptide hydrogels as a suitable scaffold-based model for the culture of the DAOY and ONS76 cell lines^53–55^. Singh et al. also described the growth of DAOY single cells on a cellulose-derived hydrogel^56^. In our study, we aimed to adopt a brain-specific matrix to provide a more tissue-realistic in vitro model of medulloblastoma metastatic dissemination. As HA is the major constituent and backbone of the brain ECM^31,32^, we utilised the HyStem HA hydrogel kit. One of the main advantages of this system is the ability to adjust the hydrogel composition concentrations to recapitulate the stiffness of the cerebellum^57^. We also experienced no issues with assay reproducibility or batch variability.

The observed differences in migratory phenotype could be reflected in the clinical features of the cell lines. UW228-3 cells were derived from a non-metastatic tumour and although they are *TP53* mutated, which classifies them as higher risk, their migratory behaviour suggests they are not an aggressive cell line^58^. DAOY cells were also obtained from a non-metastatic tumour; however, following intracerebral inoculation in nude mice, the cells were capable of invading the brain parenchyma as well as spreading over the surface and into the ventricles^59^. In addition, the majority of medulloblastoma migration and invasion in vitro experiments have been conducted with this cell line, again highlighting its ability to disseminate (reviewed by Grotzer, Neve and Baumgartner^60^). The ONS76 cell line was derived from a tumour with metastatic disease (M2 stage), indicating that these cells had metastatic potential^61^.

Differential gene expression analysis of the 3D spheroid migration model identified the upregulation of cell movement genes and the downregulation of cell cycle processes. This finding confirms that the phenotype we are observing is also reflected in transcriptional changes within the cells. We consider this a model of the “go or grow” mechanism, whereby there is a trade-off between proliferative and migratory phenotypes^62,63^. Further evaluation of the functional relevance of the transcriptional changes in the 3D spheroid migration model led us to investigate the effects of FN on cell dissemination. Although previously thought to be a tumour suppressive factor, FN plays an oncogenic role in the surrounding stromal tissues, promoting tumour progression^64^. We observed enhanced cell dissemination on HA matrices containing FN, compared to those containing COL or HA alone. Thus, our RNAseq analysis of the 3D spheroid migration model allowed us to identify a key component of the TME and demonstrated how this model could be used to understand its functional relevance.

Key to the development and use of in vitro models in cancer research is ensuring they are representative of patient disease. We combined our RNAseq analysis with publicly-available SHH medulloblastoma patient data to show that the 3D spheroid migration model recapitulates a migratory and stemness phenotype that is reflective of patient disease and prognosis. The upregulated gene list identified in this study could be used to identify those patients with a particularly poor outcome, with the Kaplan–Meier analysis showing a significant drop in overall survival within the first 2–3 years. RNAseq analysis of stemness-related genes highlighted the upregulation of Notch-related genes which were also shown to be associated with poor survival in SHH medulloblastoma cases. These markers have been previously reported for their role in SHH medulloblastomas^65–67^ and our 3D spheroid model would be ideal for future studies involving the Notch signalling pathway.

In this study, we have presented a robust and reliable 3D spheroid model of SHH medulloblastoma for the study of tumour growth, drug response, and metastatic dissemination. 3D in vitro models are not entirely new to the medulloblastoma research community. Some studies have relied on neurosphere culture approaches, whereby clusters of cells are grown in suspension, producing spheres of varying sizes and shapes, making their applicability to growth assays and drug screening studies limited^38,39,68^. Ivanov et al. used UW228-3 3D spheroids in co-culture with human neural stem cells and demonstrated their use in drug delivery assessment^14,15^. In addition, the Baumgartner group have utilised medulloblastoma 3D spheroids to quantify cell motility^17^ and have also used ex vivo cerebellar brain slice culture systems to study cell migration^16,18^. Recently, sophisticated 3D hydrogel-based models of medulloblastoma were developed by Linke et al. which were capable of identifying subgroup differences and ECM subtypes predictive of patient outcome^37^. We provide an additional 3D in vitro model which we hope will be widely adopted by fellow researchers as not only has it been well-optimised, but it is also cost-effective and simple to set up and maintain. Despite the advantages of adopting a simplistic in vitro model, the main limitation of this study is a lack of tumour complexity in relation to the TME, which consists of many different cell types in addition to tumour cells, including fibroblasts, immune cells, pericytes and ECM^69^. The TME influences each stage of tumorigenesis, from initiation to progression and metastasis. Ultimately, it will be important to validate the key findings derived from this model in more complex in vivo models to fully investigate the clinical impact of these results.

There is a huge potential for this 3D spheroid model to be expanded to include additional TME components, including astrocytes, leptomeningeal cells, macrophages, and other ECM components, growth factors or scaffolds^18,70,71^. By starting with a robust, established 3D spheroid model, other researchers have the flexibility to test primary cells, as well as customising it to build more complex models depending on their interests and experimental questions. In conclusion, our 3D spheroid model of SHH medulloblastoma is a significant improvement over standard in vitro culture techniques and is reflective of patient disease and outcome.

## Methods

### Cell lines and standard culture conditions

DAOY and D283 cell lines were obtained from ATCC (Manassas, USA), ONS76 and ONS76 dnp53 from Annette Künkele (Charité Universitätsmedizin, Berlin, Germany), UW228-3 and D458 from John R. Silber (University of Washington, Seattle, USA), HD-MD03 from Till Milde (DKFZ, Heidelberg, Germany), D425 from Marcel Kool (DKFZ, Heidelberg, Germany), and CHLA-01-MED and CHLA-01R-MED from Geoff Pilkington (University of Portsmouth, UK). DAOY, D283, D425, and D458 cells were cultured in DMEM with 10% FBS. UW228-3 cells in DMEM/F-12 with 15% FBS and 1% sodium pyruvate. ONS76, ONS76 dnp53, and HD-MB03 cells were cultured in RPMI 1640 WITH 10% FBS. CHLA-01-MED and CHLA-01R-MED were cultured in DMEM/F-12 supplemented with 2% B-27, 20 ng/mL EGF, and 20 ng/mL bFGF.

### 3D spheroid generation and culture

Cell lines were grown in their standard culture conditions, harvested, and dissociated into single cell suspensions for spheroid generation. Cells were seeded in their optimal conditions in ultra-low attachment (ULA) round-bottom 96-well plates (Corning, 7007) in a volume of 200 µL/well in universal neurosphere culture medium (DMEM/F-12, 2% B-27 supplement, 1% N-2 supplement, 2 µg/mL Heparin, 20 ng/mL EGF, and 10 ng/mL bFGF, made fresh weekly). The outer wells of the ULA plate were filled with HBSS to reduce the effects of evaporation. Plates were incubated at 37 °C in 5% CO_2_ and high humidity and spheroids were maintained by performing 50% medium replenishments every 3–4 days.

### 3D spheroid analysis

Spheroid images were taken on a camera (Canon, DS126431) attached to a brightfield microscope (Olympus, CKX41) at 10X magnification. The scale of images was determined using a calibration slide. A macro developed by Ivanov et al.^14^, compatible with the open-access software ImageJ (Fiji), was used for spheroid size analysis. The macro automatically calculated spheroid area and drew a blue outline of the detected spheroid. When the macro failed to detect the correct spheroid circumference, manual outlining was performed. Spheroid area (A) measurements were exported to Microsoft Excel and additional dimensions were calculated, including:

$${\text{Radius }}\left( {\text{r}} \right) \, = \sqrt {\frac{{\text{A}}}{{\uppi }}} \quad {\text{Diameter }}\left( {\text{d}} \right) \, = {\text{ 2r}}\quad {\text{Volume}}\,({\text{v) = }}\frac{{4}}{{3}}{\pi r}^{{3}}$$

To calculate the coefficient of variation (CV), day 4 spheroids were imaged, analysed, and variability in spheroid diameter was calculated using the following equation:

$${\text{CV }}\left( {{\%}} \right){ = }\frac{{\text{Standard deviation}}}{{{\text{Mean}}}} \times {100}$$

### Immunohistochemistry

Day 7, 14, and 21 spheroids were pooled into separate 1.5 mL microcentrifuge tubes, medium was removed and spheroids were washed with 1 mL of HBSS. The wash was removed and spheroids were resuspended in 500 µL of HistoGel (prewarmed to 60 °C). Spheroids in HistoGel were transferred to a Tissue-Tek cryomold and were rapidly cooled by placing on ice. Spheroid blocks were transferred from the cryomold into tissue processing cassettes, fixed in 4% paraformaldehyde for two hours, washed twice for 10 min in PBS, and then processed. Samples were dehydrated by incubating in a series of alcohol solutions with increasing concentrations (one bath of 50%, 70%, 90%, and four baths of 100% methanol; one hour each), cleared with xylene (three baths; one hour each), and infiltrated with molten paraffin (two baths; two hours each; vacuum on). The following day, spheroid blocks were embedded in paraffin wax before being sectioned into 4 µm sections. Spheroid sections were stained with primary antibodies against Ki67 (Cell Signalling Technology, 9449; 1:400) and CA9 (Absolute Antibody, Ab00414-1.1; 1:800) in combination with a goat anti-mouse secondary antibody (Abcam, ab214879). Slides were counterstained with Harris Modified haematoxylin and mounted before imaging.

### Drug response studies

Drug response was assessed in cell lines grown as 2D monolayers and 3D spheroids. For 2D monolayer drug response analysis, cells were harvested when they reached approximately 70% confluence and seeded at a density of 1000 cells/well in black-walled 96-well plates. Cells were left to adhere overnight before drug treatment. For 3D spheroid drug response analysis, spheroids were generated in black-walled 96-well ULA plates (Corning, 4520) to avoid transfer after treatment. Spheroids were drug treated on day 4. At the point of drug treatment, 50% of medium was removed from each well and replaced with media containing drug at 2 × concentration. Following 72 h of drug treatment, CellTiter-Glo 3D reagent (Promega) was added to each well at a 1:1 ratio. The plates were shaken at 250 rpm for 5 min in an orbital incubator to promote cell lysis, followed by an additional 25 min incubation at room temperature. Luminescence was measured on a FLUOstar Omega plate reader. Drug response was calculated as a percentage of the vehicle-treated control and dose–response curves and IC_50_ values were generated by non-linear regression analysis.

3D spheroid drug response was also assessed by measuring changes in spheroid volume. To do this, cells were seeded on day 0 and the resulting 3D spheroids were drug treated on day 4 for 72 h. A complete medium replacement was not performed after treatment as manipulation to the spheroids resulted in cell loss and disruption. Instead 50% medium replenishment, resulting in a 1:2 dilution of the remaining drug and residual drug activity, was performed as is considered acceptable in spheroid-based drug assays^9^. To measure changes in spheroid volume, images were taken pre-treatment (day 4), during treatment (days 5 and 6), and post-treatment (days 7, 10 and 13). Significant differences in volume between the vehicle- and drug-treated spheroids were calculated using repeated measures two-way ANOVA analyses with Dunnett’s multiple comparisons post-hoc test.

### Hyaluronan hydrogel migration models

Hyaluronan hydrogels were prepared according to the manufacturer’s instructions (HyStem hydrogel kit; ESI-BIO, GS311). A hydrogel with a stiffness around 1.5–2 kPa, within the range of normal brain tumour stiffness^72^, was produced by making a 1% Glycosil (thiol-modified HA) and 2% ExtraLink-Lite (PEGDA) solution. Collagen I (Cultrex, 3447-020-01), and fibronectin (Cultrex, 3420-001-03) were added to hyaluronan hydrogels at a concentration of 50 µg/mL. For HyStem migration experiments, 50 µL of diluted hydrogel was added to each well of a tissue-culture treated 96-well plate and the solution was spread evenly over the well’s surface. The hydrogel-coated plates were incubated at 37 °C for 30 min to allow the gel layer to set. Day 4 spheroids were then transferred from the 96-well ULA plate in which they were generated, to the hydrogel-coated plate in 100 µL of neurosphere medium. An additional 100 µL of fresh neurosphere medium was added to each well and plates were incubated at 37 °C for 1 h to allow the spheroids to settle on the matrix. Spheroid migration across HyStem hydrogels was assessed by manual imaging over a 72 h period. Relative spheroid outgrowth was calculated by measuring the area covered by migrating cells and normalising to the initial spheroid size at 0 h. Significant differences in relative spheroid outgrowth were calculated using two-way ANOVA analyses with Tukey’s multiple comparisons post-hoc test.

### Next-generation sequencing

Ten wells per condition were harvested and pooled into a 1.5 mL microcentrifuge tube and snap-frozen in liquid nitrogen and stored at −80 °C. RNA isolation was performed using the NucleoSpin RNA Plus kit (Macherey–Nagel, 740984). Samples were transferred to tubes containing lysing Matrix D beads (MP Biomedicals, 11412420), 1.4 mm ceramic spheres used for grinding matrices. 350 µL of lysis buffer was added to each tube and samples were homogenised for 30 s using a Fast-Prep24 tissue homogeniser (MP Biomedicals, 12079310). Samples were then transferred to a new 1.5 mL microcentrifuge tube and centrifuged at 11,000×*g* for 5 min to pellet the hydrogel at the bottom of the tube. The rest of the extraction was performed according to the RNA isolation kit manufacturer’s instructions. Next-generation sequencing (NGS) was performed by QIAGEN Genomic Services (Hilden, Germany). More details regarding the NGS technologies adopted are included in the Supplementary Methods S1.

### Patient dataset analysis

K-means clustering and Kaplan–Meier survival analysis was performed on the R2 Genomics Analysis and Visualization Platform (http://r2.amc.nl) using the Cavalli medulloblastoma dataset^4^.

## Supplementary Information

Supplementary Information 1.

Supplementary Information 2.

## Data Availability

The RNA sequencing data have been deposited in the ArrayExpress database at EMBL-EBI (http://www.ebi.ac.uk/arrayexpress) under accession number E-MTAB-10127.

## References

[1] Pizer BL, Clifford SC (2009). The potential impact of tumour biology on improved clinical practice for medulloblastoma: Progress towards biologically driven clinical trials. Br. J. Neurosurg..

[2] Northcott PA (2012). Medulloblastomics: The end of the beginning. Nat. Rev. Cancer.

[3] Taylor MD (2012). Molecular subgroups of medulloblastoma: The current consensus. Acta Neuropathol..

[4] Cavalli FMG (2017). Intertumoral heterogeneity within medulloblastoma subgroups. Cancer Cell.

[5] Schwalbe EC (2017). Novel molecular subgroups for clinical classification and outcome prediction in childhood medulloblastoma: a cohort study. Lancet Oncol..

[6] Ramaswamy V (2016). Risk stratification of childhood medulloblastoma in the molecular era: The current consensus. Acta Neuropathol..

[7] Packer, R. J., Macdonald, T., Vezina, G., Keating, R. & Santi, M. Medulloblastoma and primitive neuroectodermal tumors. in *Handbook of Clinical Neurology* **105**, 529–548 (Elsevier, 2012). 10.1016/B978-0-444-53502-3.00007-0 22230517

[8] Sutherland RM, McCredie JA, Rodger W (1971). Growth of multicell spheroids in tissue culture as a model of nodular carcinomas. J. Natl. Cancer Inst..

[9] Friedrich J, Seidel C, Ebner R, Kunz-Schughart LA (2009). Spheroid-based drug screen: Considerations and practical approach. Nat. Protoc..

[10] Vinci M (2012). Advances in establishment and analysis of three-dimensional tumor spheroid-based functional assays for target validation and drug evaluation. BMC Biol..

[11] Zanoni M (2016). 3D tumor spheroid models for in vitro therapeutic screening: A systematic approach to enhance the biological relevance of data obtained. Sci. Rep..

[12] Mittler F (2017). High-content monitoring of drug effects in a 3D spheroid model. Front. Oncol..

[13] Vinci, M., Box, C., Zimmermann, M. & Eccles, S. A. Tumor spheroid-based migration assays for evaluation of therapeutic agents. in *Target Identification and Validation in Drug Discovery* **986**, 253–266 (Springer Protocols, 2013). 10.1007/978-1-62703-311-4_16 23436417

[14] Ivanov DP (2014). Multiplexing spheroid volume, resazurin and acid phosphatase viability assays for high-throughput screening of tumour spheroids and stem cell neurospheres. PLoS ONE.

[15] Ivanov DP (2015). In vitro co-culture model of medulloblastoma and human neural stem cells for drug delivery assessment. J. Biotechnol..

[16] Neve A, Kumar KS, Tripolitsioti D, Grotzer MA, Baumgartner M (2017). Investigation of brain tissue infiltration by medulloblastoma cells in an ex vivo model. Sci. Rep..

[17] Kumar KS (2015). Computer-assisted quantification of motile and invasive capabilities of cancer cells. Sci. Rep..

[18] Kumar KS (2018). TGF-β determines the pro-migratory potential of bFGF signaling in medulloblastoma. Cell Rep..

[19] Schönholzer MT (2020). Real-time sensing of MAPK signaling in medulloblastoma cells reveals cellular evasion mechanism counteracting dasatinib blockade of ERK activation during invasion. Neoplasia (United States).

[20] Ivascu A, Kubbies M (2007). Diversity of cell-mediated adhesions in breast cancer spheroids. Int. J. Oncol..

[21] Sittampalam, G. S., Grossman, A., Brimacombe, K., Arkin, M. & Auld, D. HTS assay validation. in *Assay Guidance Manual* 945–969 (Eli Lilly & Company and the National Center for Advancing Translational Sciences, 2004). 22553862

[22] Bonfim-Silva, R. *et al.* Biological characterization of the UW402, UW473, ONS-76 and DAOY pediatric medulloblastoma cell lines. *Cytotechnology* 1–11 (2019). doi:10.1007/s10616-019-00332-3 10.1007/s10616-019-00332-3 PMC6787134 31346954

[23] Dimasi JA, Feldman L, Seckler A, Wilson A (2010). Trends in risks associated with new drug development: Success rates for investigational drugs. Clin. Pharmacol. Ther..

[24] Zhukova N (2013). Subgroup-specific prognostic implications of TP53 mutation in medulloblastoma. J. Clin. Oncol..

[25] Ramaswamy V (2016). Medulloblastoma subgroup-specific outcomes in irradiated children: Who are the true high-risk patients?. Neuro. Oncol..

[26] Gottlieb A (2017). RITA displays anti-tumor activity in medulloblastomas independent of TP53 status. Oncotarget.

[27] Szakács G, Paterson JK, Ludwig JA, Booth-Genthe C, Gottesman MM (2006). Targeting multidrug resistance in cancer. Nat. Rev. Drug Discov..

[28] Jackson, D. V, Sethi, V. S., Spurr, C. L. & McWhorter, J. M. Pharmacokinetics of vincristine in the cerebrospinal fluid of humans. *Cancer Res.* **41**, 1466–1468 (1981). 6260340

[29] Othman RT (2014). Overcoming multiple drug resistance mechanisms in medulloblastoma. Acta Neuropathol. Commun..

[30] Fults DW, Taylor MD, Garzia L (2019). Leptomeningeal dissemination: a sinister pattern of medulloblastoma growth. J. Neurosurg. Pediatr..

[31] Rauch U (2004). Extracellular matrix components associated with remodeling processes in brain. Cell. Mol. Life Sci..

[32] Rauch U (2007). Brain matrix: structure, turnover and necessity. Biochem. Soc. Trans..

[33] Zhang X (2015). Notch1 induces epithelial-mesenchymal transition and the cancer stem cell phenotype in breast cancer cells and STAT3 plays a key role. Int. J. Oncol..

[34] Jin M, Gao D, Wang R, Sik A, Liu K (2020). Possible involvement of TGF-ß SMAD-mediated epithelial-mesenchymal transition in pro-metastatic property of PAX6. Oncol. Rep..

[35] Lou X (2013). SOX2 targets fibronectin 1 to promote cell migration and invasion in ovarian cancer: New molecular leads for therapeutic intervention. Omi. A J. Integr. Biol..

[36] Tripolitsioti D (2018). MAP4K4 controlled integrin β1 activation and c-Met endocytosis are associated with invasive behavior of medulloblastoma cells. Oncotarget.

[37] Linke F (2020). 3D hydrogels reveal medulloblastoma subgroup differences and identify extracellular matrix subtypes that predict patient outcome. J. Pathol..

[38] da Silva PBG (2017). High OCT4A levels drive tumorigenicity and metastatic potential of medulloblastoma cells. Oncotarget.

[39] Kaid C (2015). miR-367 promotes proliferation and stem-like traits in medulloblastoma cells. Cancer Sci..

[40] Del Duca D, Werbowetski T, Del Maestro RF (2004). Spheroid preparation from hanging drops: Characterization of a model of brain tumor invasion. J. Neurooncol..

[41] Gayan S, Teli A, Dey T (2017). Inherent aggressive character of invasive and non-invasive cells dictates the in vitro migration pattern of multicellular spheroid. Sci. Rep..

[42] Ivascu A, Kubbies M (2006). Rapid generation of single-tumor spheroids for high-throughput cell function and toxicity analysis. J. Biomol. Screen..

[43] Leung BM, Lesher-Perez SC, Matsuoka T, Moraes C, Takayama S (2015). Media additives to promote spheroid circularity and compactness in hanging drop platform. Biomater. Sci..

[44] Carlsson J, Acker H (1988). Relations between pH, oxygen partial pressure and growth in cultured cell spheroids. Int. J. Cancer.

[45] Grimes DR, Fletcher AG, Partridge M (2014). Oxygen consumption dynamics in steady-state tumour models. R. Soc. Open Sci..

[46] Hirschhaeuser F (2010). Multicellular tumor spheroids: An underestimated tool is catching up again. J. Biotechnol..

[47] Wojtkowiak JW, Verduzco D, Schramm KJ, Gillies RJ (2011). Drug resistance and cellular adaptation to tumor acidic pH microenvironment. Mol. Pharm..

[48] Swietach P, Hulikova A, Patiar S, Vaughan-Jones RD, Harris AL (2012). Importance of intracellular pH in determining the uptake and efficacy of the weakly basic chemotherapeutic drug, doxorubicin. PLoS ONE.

[49] Cheung-Ong K, Giaever G, Nislow C (2013). DNA-damaging agents in cancer chemotherapy: Serendipity and chemical biology. Chem. Biol..

[50] Vanner RJ (2014). Quiescent Sox2+ cells drive hierarchical growth and relapse in Sonic Hedgehog subgroup medulloblastoma. Cancer Cell.

[51] Meng W, Garnett MC, Walker DA, Parker TL (2016). Penetration and intracellular uptake of poly(glycerol-adipate) nanoparticles into three-dimensional brain tumour cell culture models. Exp. Biol. Med..

[52] Tchoryk A (2019). Penetration and uptake of nanoparticles in 3D tumor spheroids. Bioconjug. Chem..

[53] Altunbas A, Lee SJ, Rajasekaran SA, Schneider JP, Pochan DJ (2011). Encapsulation of curcumin in self-assembling peptide hydrogels as injectable drug delivery vehicles. Biomaterials.

[54] Worthington P (2017). Beta-hairpin hydrogels as scaffolds for high-throughput drug discovery in three-dimensional cell culture. Anal. Biochem..

[55] Worthington P (2019). Implementation of a high-throughput pilot screen in peptide hydrogel-based three-dimensional cell cultures. SLAS Discov..

[56] Singh T, Kothapalli C, Varma D, Nicoll SB, Vazquez M (2014). Carboxymethylcellulose hydrogels support central nervous system-derived tumor-cell chemotactic migration: comparison with conventional extracellular matrix macromolecules. J. Biomater. Appl..

[57] Vanderhooft JL, Alcoutlabi M, Magda JJ, Prestwich GD (2009). Rheological properties of cross-linked hyaluronan-gelatin hydrogels for tissue engineering. Macromol. Biosci..

[58] Keles GE (1995). Establishment and characterization of four human medulloblastoma-derived cell lines. Oncol. Res..

[59] Jacobsen PF, Jenkyn DJ, Papadimitriou JM (1985). Establishment of a human medulloblastoma cell line and its heterotransplantation into nude mice. J. Neuropathol. Exp. Neurol..

[60] Grotzer MA, Neve A, Baumgartner M (2016). Dissecting brain tumor growth and metastasis in vitro and ex vivo. J. Cancer Metastasis Treat..

[61] Tamura K (1989). Expression of major histocompatibility complex on human medulloblastoma cells. Cancer Res..

[62] Biddle A (2011). Cancer stem cells in squamous cell carcinoma switch between two distinct phenotypes that are preferentially migratory or proliferative. Cancer Res..

[63] Garay T (2013). Cell migration or cytokinesis and proliferation? Revisiting the ‘go or grow’ hypothesis in cancer cells in vitro. Exp. Cell Res..

[64] Lin TC (2019). Fibronectin in cancer: Friend or foe. Cells.

[65] Kahn SA (2018). Notch1 regulates the initiation of metastasis and self-renewal of Group 3 medulloblastoma. Nat. Commun..

[66] Fiaschetti G (2014). NOTCH ligands JAG1 and JAG2 as critical pro-survival factors in childhood medulloblastoma. Acta Neuropathol. Commun..

[67] Natarajan S (2013). Notch1-induced brain tumor models the sonic hedgehog subgroup of human medulloblastoma. Cancer Res..

[68] Zanini C (2013). Medullospheres from DAOY, UW228 and ONS-76 cells: Increased stem cell population and proteomic modifications. PLoS ONE.

[69] Wang M (2017). Role of tumor microenvironment in tumorigenesis. J. Cancer.

[70] Margol AS (2015). Tumor-associated macrophages in SHH subgroup of medulloblastomas. Clin. Cancer Res..

[71] Liu Y (2017). Astrocytes promote medulloblastoma progression through hedgehog secretion. Cancer Res..

[72] Barnes JM, Przybyla L, Weaver VM (2017). Tissue mechanics regulate brain development, homeostasis and disease. J. Cell Sci..

