# Supplementary material for: 3D spheroid models of paediatric SHH medulloblastoma mimic tumour biology, drug response and metastatic dissemination
Source: Sci Rep. 2021 Feb 19;11:4259. doi: 10.1038/s41598-021-83809-6 (PMC7895940; doi:10.1038/s41598-021-83809-6)
Supplement: Supplementary file 2 — Supplementary Information 2. [file 41598_2021_83809_MOESM2_ESM.docx]

**3D spheroid models of paediatric SHH medulloblastoma mimic tumour biology, drug response and metastatic dissemination**

**Sophie J. Roper^1^, Franziska Linke^1^, Paul J. Scotting^2^, and Beth Coyle^1^***

**Supplementary information**

**
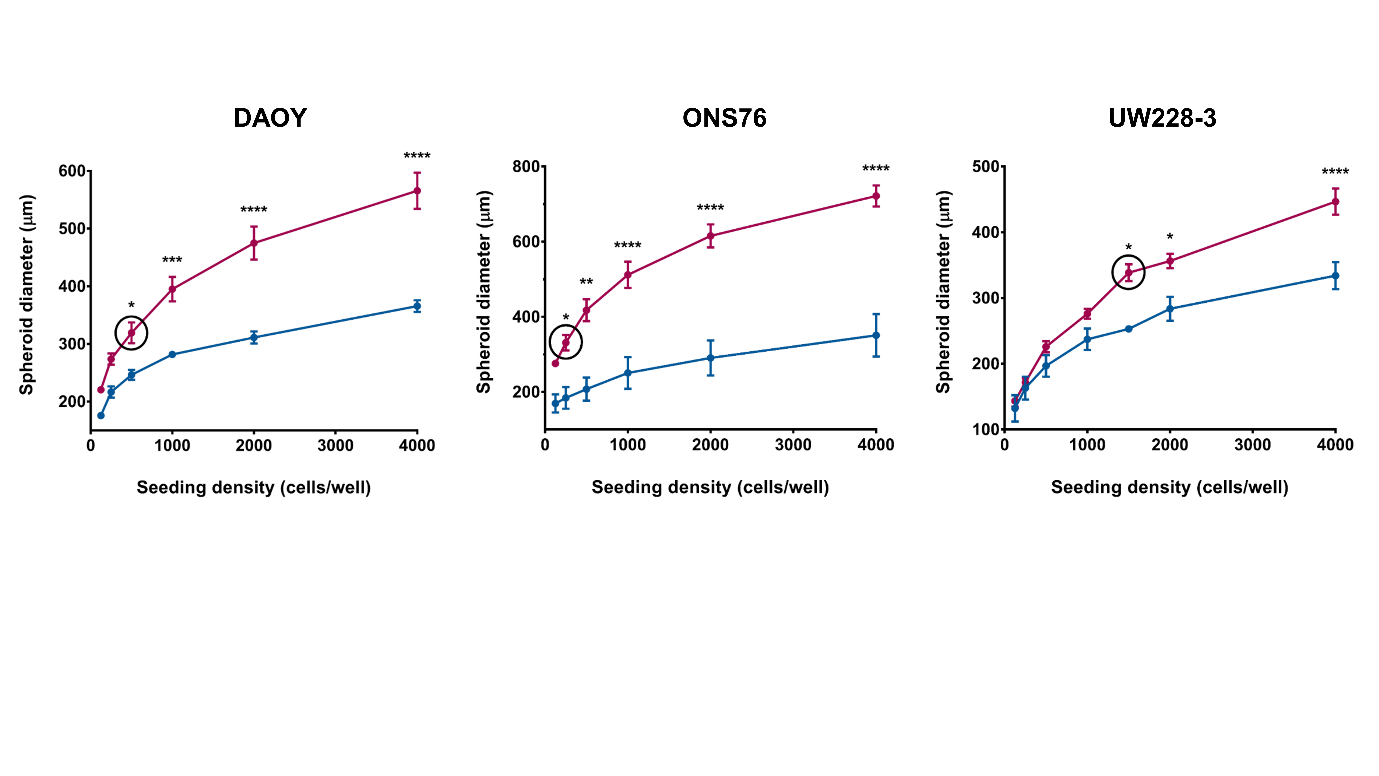
**

**Supplementary Figure S1. Relationship between cell seeding density (cells/well) and spheroid diameter (µm) after four days growth.** 3D spheroids generated in neurosphere medium (red) were significantly larger than those cultured in standard medium (blue) at selected seeding densities. 500, 250, and 1,500 cells/well were chosen as optimal seeding densities for DAOY, ONS76, and UW228-3 cell lines, respectively (circled), to generate 3D spheroids with a diameter of 250-350 µm by day 4. Error bars represent the mean ± SEM of n=3 experiments each containing 3 replicates. Significance was calculated using two-way ANOVA analyses with Sidak’s multiple comparisons post-hoc test (*p≤0.05, ***p≤0.001, ****p≤0.0001).

**
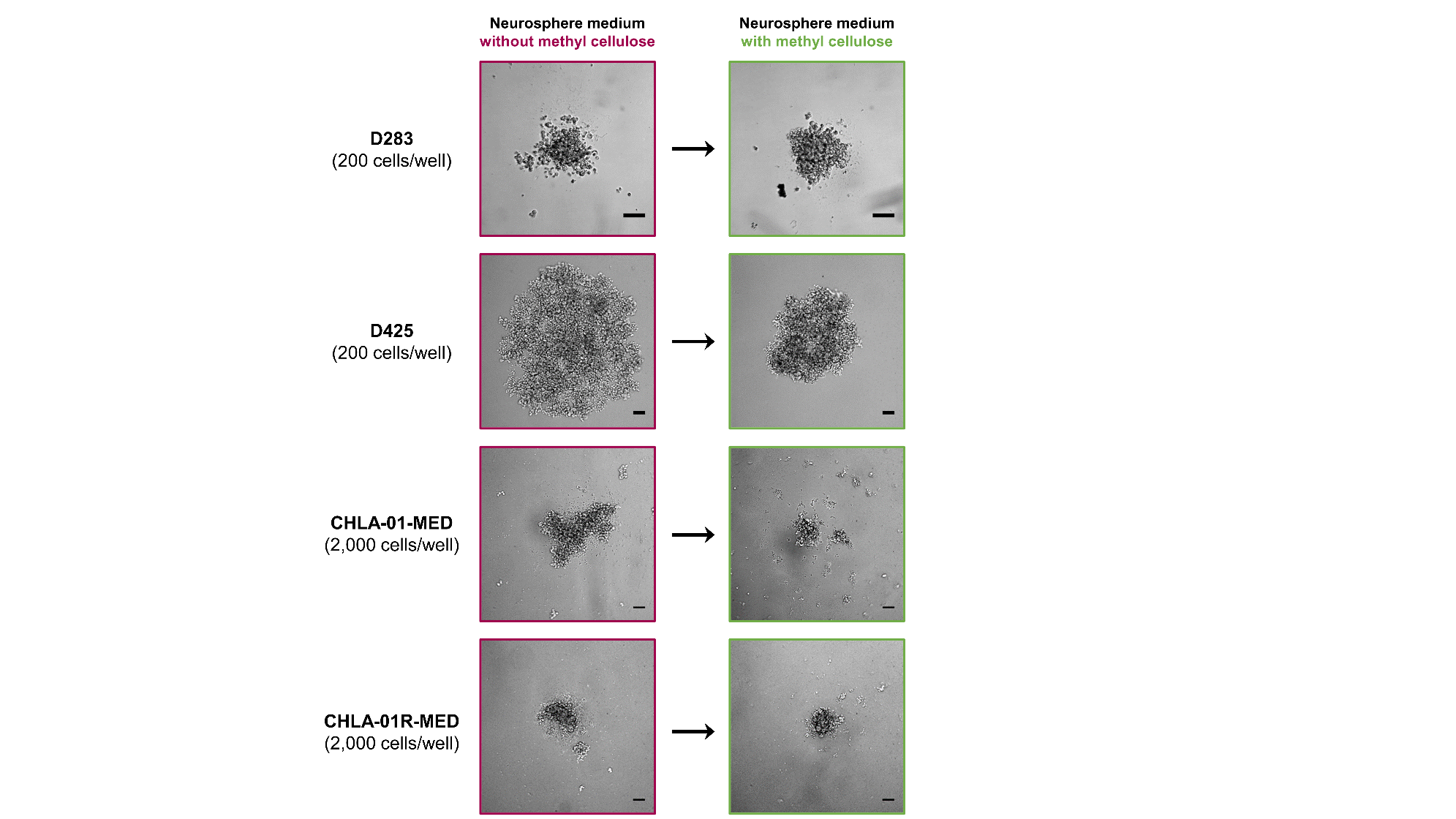
**

**Supplementary Figure S2. Examples of additional medulloblastoma cell lines which were tested for use in the 3D spheroid model.** Group 3 cell lines (D283 and D425) and Group 4 cell lines (CHLA-01-MED and CHLA-01R-MED) were seeded at 200 cells/well and 2,000 cells/well, respectively, in neurosphere medium with or without 0.24% methyl cellulose. All of these cell lines were deemed unsuitable for 3D spheroid culture in these conditions as they did not produce one single, tight aggregate per well.

**
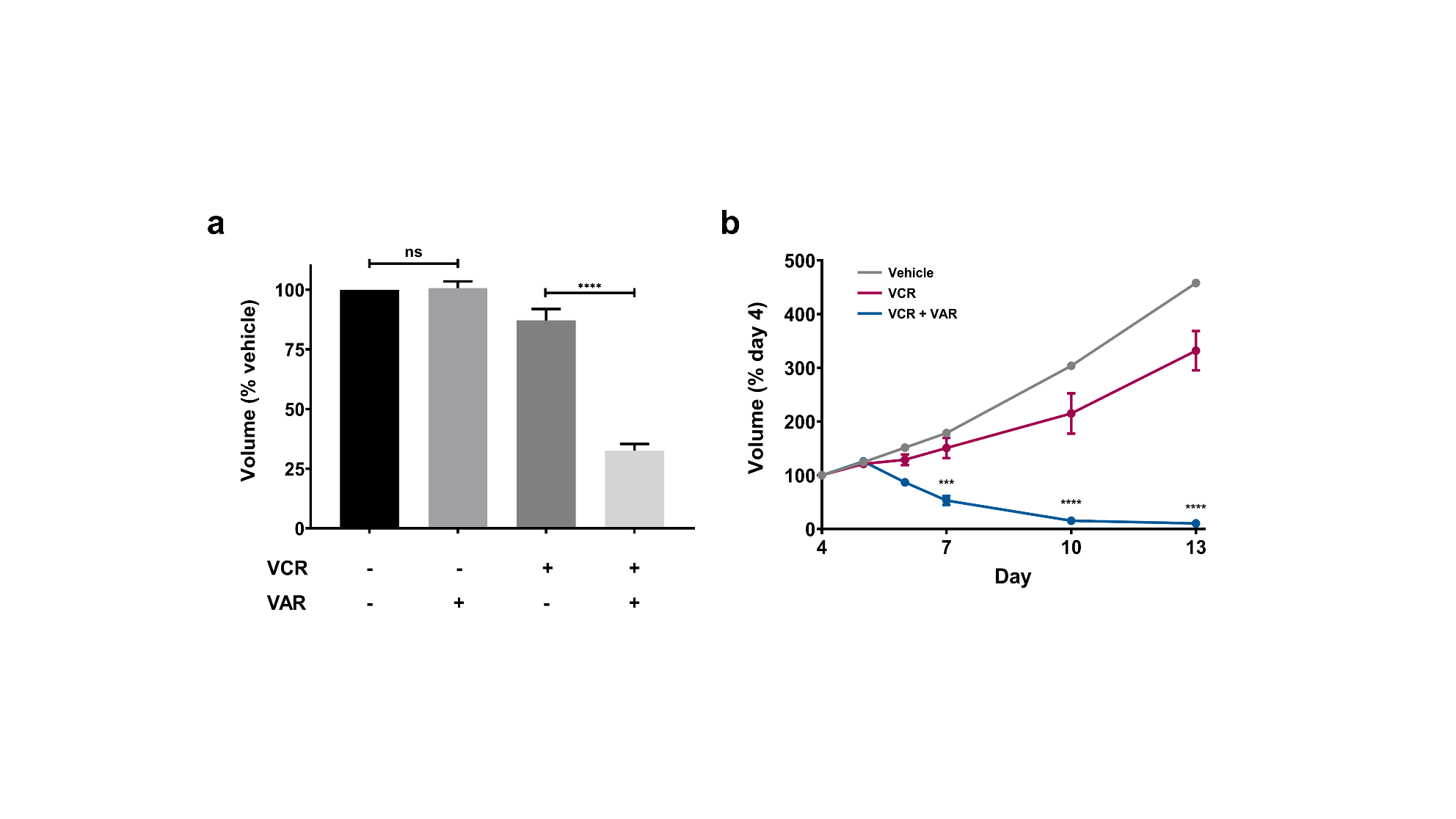
**

**Supplementary Figure S3. Potentiation of vincristine response with vardenafil in DAOY 3D spheroids.** (**a**) DAOY 3D spheroids were treated with vincristine (VCR; 1 nM) in combination with vardenafil (VAR; 10 µM) for 72 hours. Images were taken immediately after treatment to measure changes in spheroid volume relative to the vehicle-treated control. There was no difference in volume after vardenafil-only treatment. Vincristine efficacy could be potentiated when treated in combination with vardenafil. Error bars represent the mean ± SEM of n≥3 experiments each containing 3 replicates. Significance was calculated using one-way ANOVA analyses with Sidak’s multiple comparisons post-hoc test (ns=not significant, ****p≤0.0001). (**ii**) Continual monitoring of the effects of combined vincristine and vardenafil treatment (blue) showed a significant continual reduction in spheroid volume following treatment compared to vincristine alone (red). Significance differences between single and combination treatment were calculated using repeated measures two-way ANOVA analyses with Tukey’s multiple comparisons post-hoc test (***p≤0.01, ****p≤0.0001).


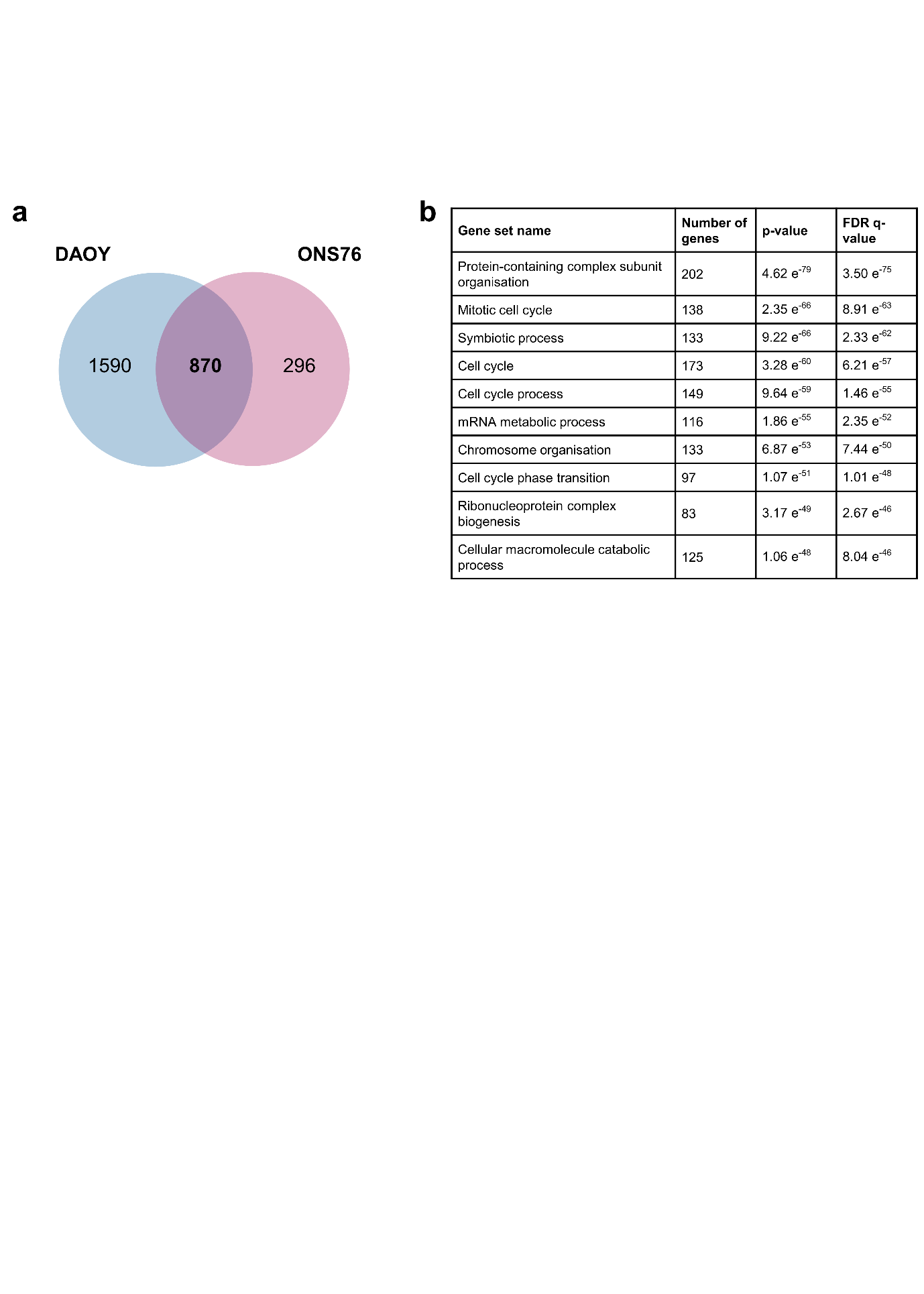


**Supplementary Figure S4. Downregulated genes in the 3D spheroid migration model.** Differential gene expression analysis was conducted to identify downregulated genes in the 3D spheroid migration model. (**a**) 870 downregulated genes (Log2 fold change ≥1) were shared between DAOY and ONS76 samples. (**b**) Top 10 Gene Ontology (GO) biological process gene sets significantly associated with these 870 downregulated genes was assessed using Gene Set Enrichment Analysis. Significances were determined using an uncorrected p-value and a q-value (a p-value adjusted using the Benjamini-Hochberg False Discovery Rate approach) to correct for multiple testing.

**
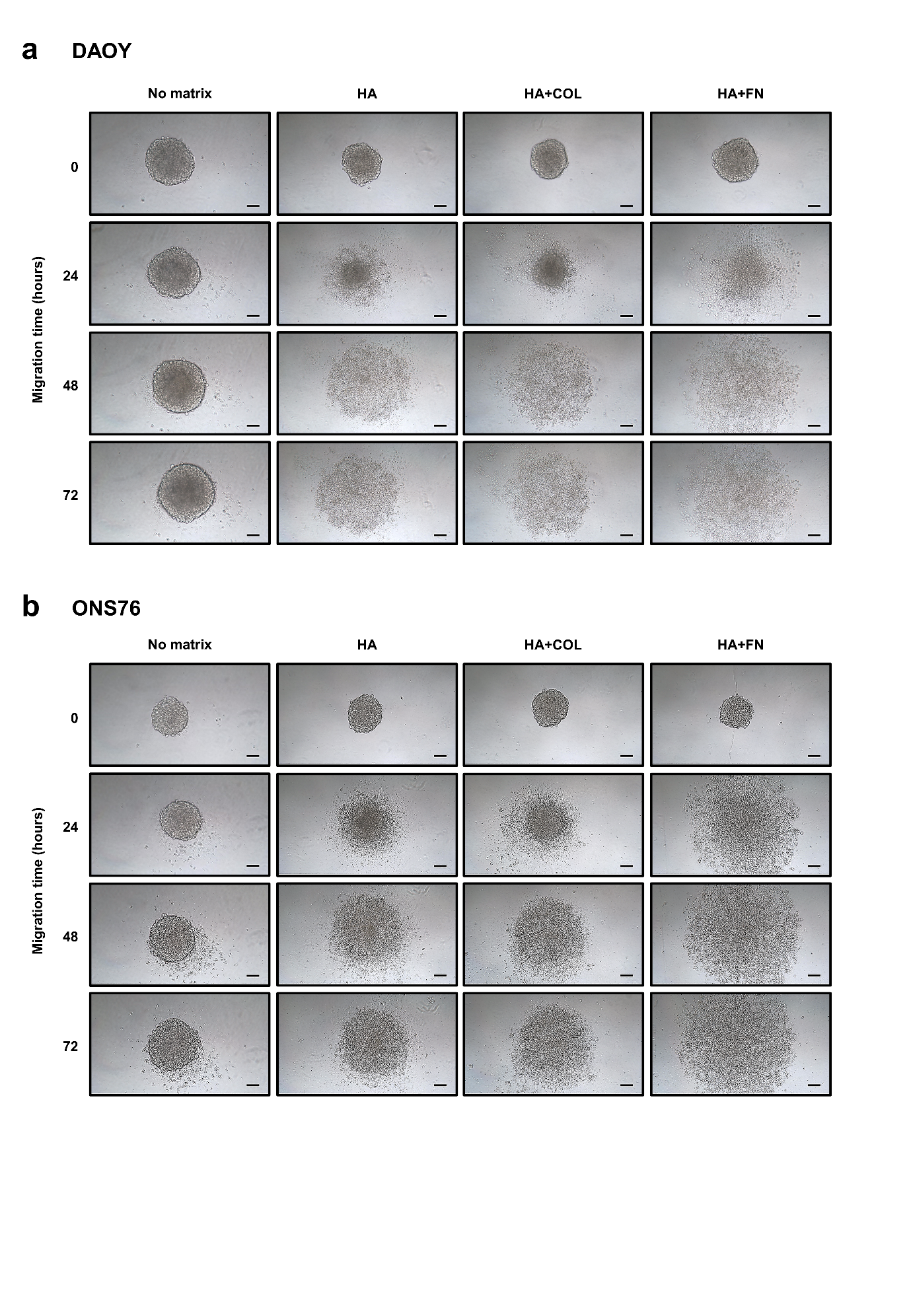
**

**Supplementary Figure S5. 3D spheroid migration across hyaluronan hydrogels.** DAOY (**a**) and ONS76 (**b**) 3D spheroids were transferred to a 96-well flat-bottom plate coated with hyaluronan (HA) matrices with or without the extracellular matrix components collagen I (COL) and fibronectin (FN) on day 4 (0 hours). A no matrix control was included to assess normal 3D spheroid growth. Cell dissemination across the matrices was assessed over a 72 hour period. Images of the same 3D spheroid at different time points are shown (scale bar: 100 µm) and are representative of 3 independent experiments each containing 3 replicates.


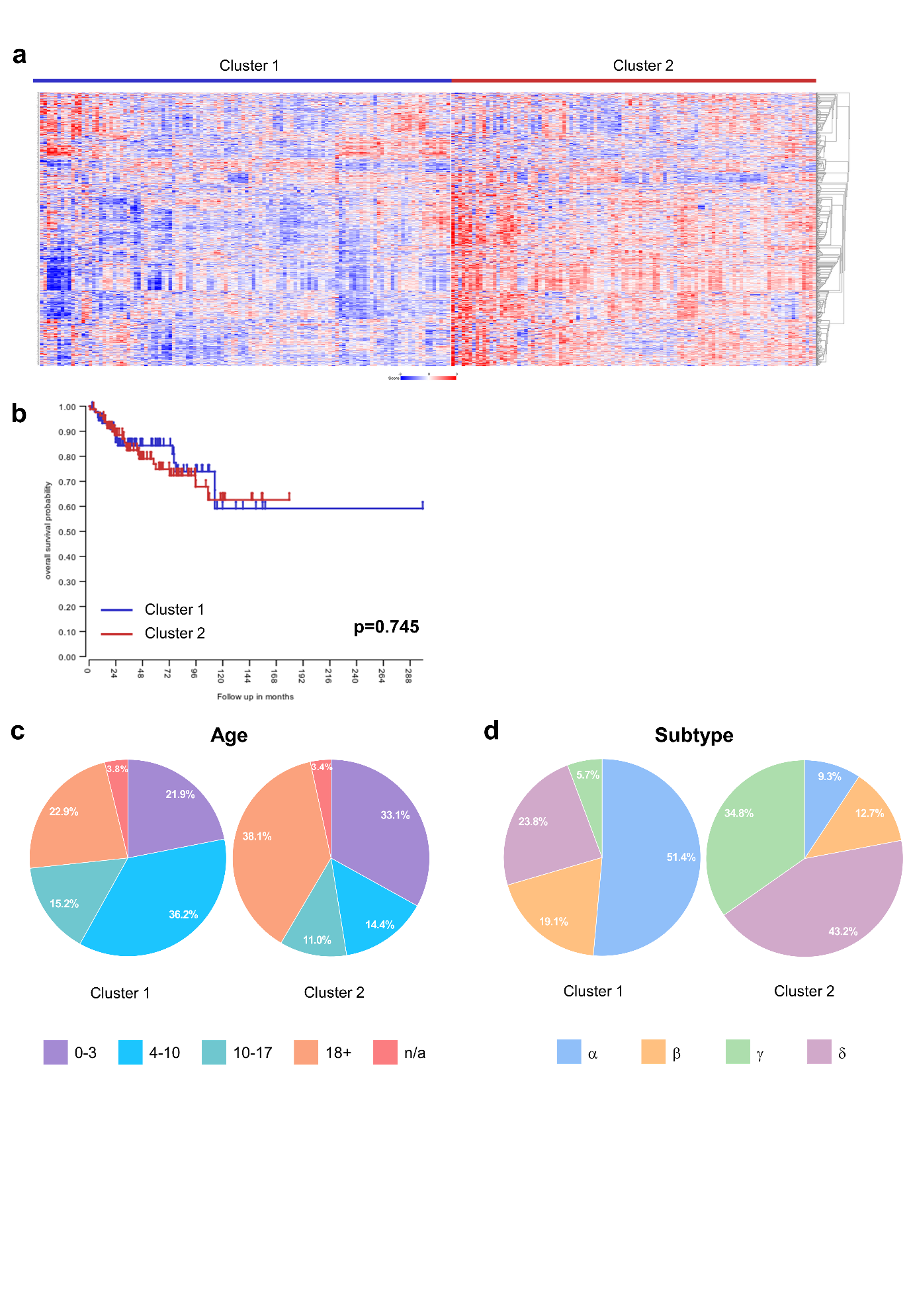


**Supplementary Figure S6. Relating downregulated genes in the 3D spheroid migration model to patient data.** The R2 Genomics Analysis and Visualization Platform was used to analyse the Cavalli medulloblastoma dataset. (**a**) Heatmap showing the *k*-means clustering of 223 SHH subgroup samples into two groups (cluster 1: n=125; cluster 2: n=98) based on the expression of the 870 downregulated genes in the 3D spheroid migration model. (**b**) Kaplan-Meier overall survival curves for clusters 1 and 2. Pie charts showing the age (**c**) and SHH subtype (**d**) distributions in clusters 1 and 2.

**
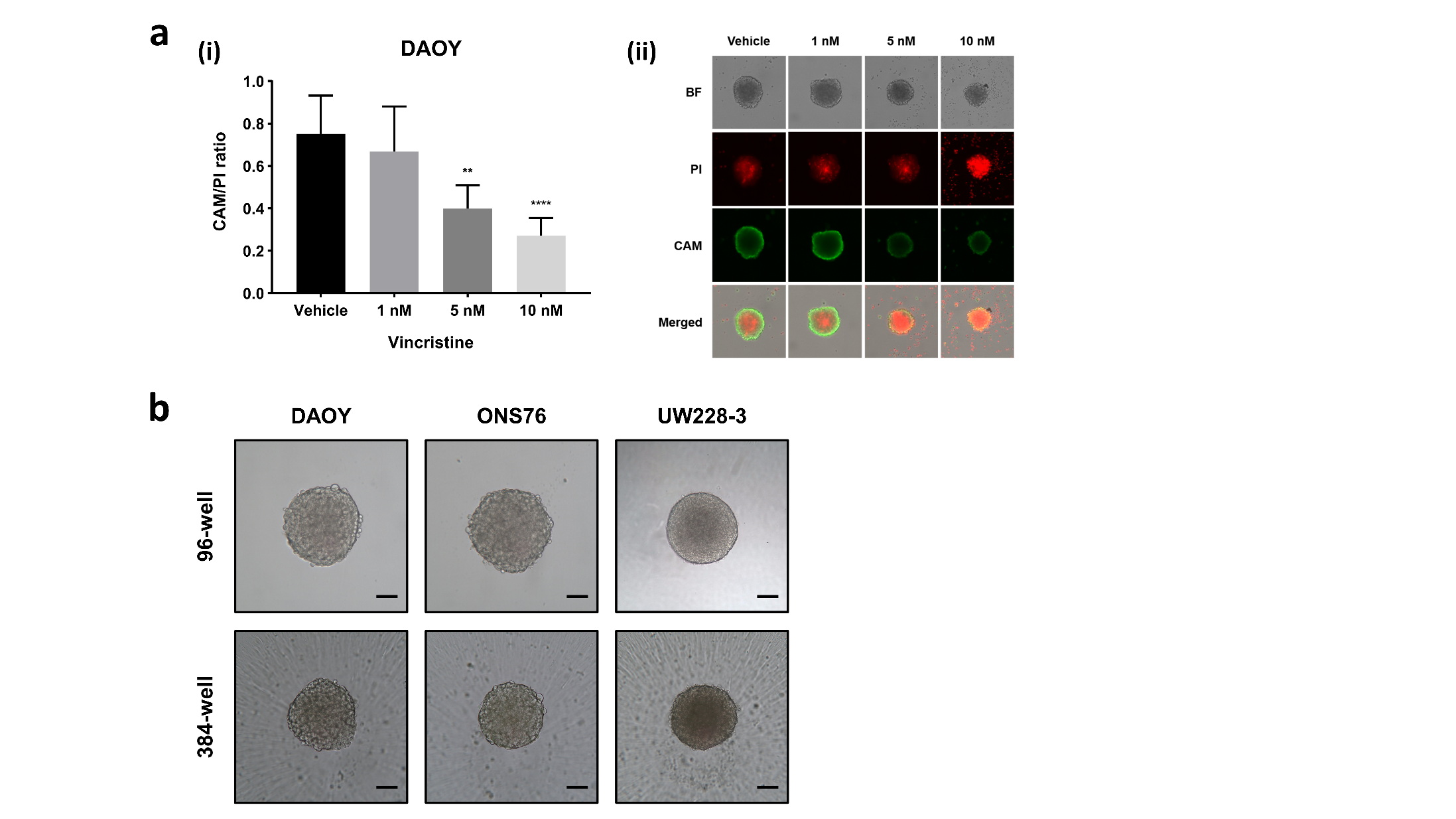
**

**Supplementary Figure S7. Exemplification of higher throughput techniques for 3D spheroid drug response analysis.** (**a**) DAOY 3D spheroids were treated with three concentrations of vincristine (1, 5, and 10 nM) for 72 hours and stained with the viability markers Calcein-AM (CAM; live cells) and Propidium Iodide (PI; dead cells). (i) Automated imaging analysis using the Celigo cytometer showed a decrease in CAM/PI ratio at increasing concentrations of vincristine, representing a decrease in viability. Error bars represent the mean ± SD of one experiment containing 6 replicates. Significance differences in CAM/PI ratios in comparison to vehicle-treated controls was calculated using one-way ANOVA analyses with Dunnett’s multiple comparisons post-hoc test (**p≤0.01, ****p≤0.0001). (ii) Representative brightfield (BF), PI, CAM, and merged images of treated spheroids are shown. (**b**) Comparison of SHH medulloblastoma 3D spheroids generated in 96-well and 384-well ultra-low attachment round-bottom plates. Representative images of day 4 spheroids of the SHH medulloblastoma cell lines (DAOY, ONS76, and UW228-3) are shown (scale bar: 100 µm).

**Supplementary Table S1. Comparison of some available *in vitro* models.**

| **Model** | **Uses** | **Advantages** | **Disadvantages** |
| --- | --- | --- | --- |
| **Spheroids**  Aggregates of cells grown in suspension or embedded within an ECM matrix. | - Growth and proliferation assays^1^ - Drug screening^2^ - Cell-matrix interactions^3^ - Cell migration/invasion^4^ | - Cost-effective - Easily reproducible - High-throughput - Long-term culture possible | - Some spheroid generation methods can be difficult |
| **Scaffold-based**  Cells are embedded within a gel or matrix and the interaction of the cells with the surrounding matrix can be observed. | - Growth and proliferation assays - Cell migration/invasion - Matrix remodelling - Cell adhesion | - Long-term culture possible - Ability to choose organ-specific matrices - Ability to modify matrix stiffness to recapitulate tissue ECM | - Gels/matrices can be expensive - Imaging cells within gels can be difficult |
| **Transwell**  Can be used to assess cell movement across a porous membrane. | - Cell migration and invasion - Cell adhesion - Chemotaxis - Matrix remodelling | - Cost-effective - Accessible - Can compare metastatic potential of cells | - Imaging is difficult - Chemotactic gradients may not be sustained for long-term experiments |
| **Organotypic brain slice culture**  Involves the culturing and maintenance of cerebellar brain slices *ex vivo*, coupled with cell line co-culture. | - Growth assays - Drug screening^5^ - Penetration and uptake of nanoparticles^6^ - Cell migration^7^ | - Bridges the gap between *in vitro* and *in vivo* studies | - Expensive |

**Supplementary Table S2. Comparison of spheroid generation methods.**

| **Method** | **Uses** | **Advantages** | **Disadvantages** |
| --- | --- | --- | --- |
| **Spontaneous formation**  Utilises non-adherent surfaces, e.g. ultra-low attachment round-bottom plates, to allow the spontaneous formation of spheroids from single cells. | - Growth assays - Drug screening - Cell migration/invasion | - Simple and inexpensive method - Spheroid size highly reproducible and can be accurately controlled by adjusting cell seeding density - High-throughput: available in 96- and 384-well format - Spheroids centrally-located in each well: ideal for imaging - Long-term culture possible - Spheroids can be embedded in matrix | - Media changes with small volumes can be difficult |
| **Hanging drop**  Involves placing small drops of cells in suspension (around 15-30 µL) onto the underside of the lid of a tissue culture dish. When the lid is inverted, cells accumulate in the drop and aggregate, forming a spheroid over time. | - Growth assays - Drug screening | - Does not rely on matrices or scaffolds, and instead allows the cells to naturally aggregate - High-throughput: available in 96- and 384-well format | - Long-term culture difficult - Media changes with small volumes can be difficult |
| **Suspension culture**  Involves the continual agitation of a cell suspension within a container to promote cell-cell interactions. The two main approaches are spinner flasks (containing a stirrer which continuously moves the cell suspension) and rotary cell culture systems. | - High-throughput production of spheroids - Drug testing | - Produces a large yield of spheroids | - Agitation can alter spheroid morphology, producing aggregates of differing sizes - No individual compartments for spheroids - Requires specialised rotary systems |
| **Scaffold-based**  Where cells are plated on top of or within a matrix. | - Growth assays - Cell-matrix interactions - Cell migration/invasion | - Ability to choose organ-specific matrices - Ability to modify matrix stiffness to recapitulate tissue ECM | - Scaffolds can be expensive - Lower throughput than ULA plate and hanging-drop methods - Batch-to-batch variation |

**Supplementary Table S3. Summary of the medulloblastoma cell lines tested for their suitability in the 3D spheroid model.** Nine medulloblastoma cell lines representing three of the four molecular subgroups (SHH, Group 3, and Group 4) were tested for their suitability in the 3D spheroid model. Optimised seeding densities and any necessary modifications required for 3D spheroid culture are shown. For cell lines which formed suitable 3D spheroids, examples of their morphology on day 4 are shown.

|  | **Cell line** | **Clinical characteristics** | **Suitable for 3D spheroid culture?** | **Optimal density (cells/well)** | **Culture conditions** | **Example of day 4 spheroid**  (scale bar: 100 µm) |
| --- | --- | --- | --- | --- | --- | --- |
| **SHH** | DAOY | Non-metastatic primary tumour with *TP53* mutation of 4yo male | ✓ | 500 | NS medium | 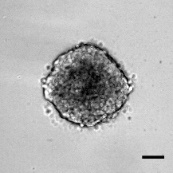 |
|  | ONS76 | *TP53*^wt^ metastatic tumour of 2yo female | ✓ | 250 | NS medium | 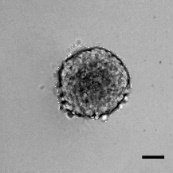 |
|  | UW228-3 | Non-metastatic primary tumour with *TP53* mutation of 9yo female | ✓ | 1,500 | NS medium | 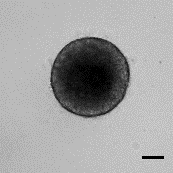 |
| **Group 3** | HD-MB03 | Metastatic tumour of 3yo female | ✓ | 200 | NS medium  + 0.24% methyl cellulose  + centrifugation | 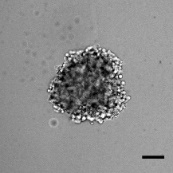 |
|  | D283 | Peritoneal metastasis of 6yo male | 🗶 | Not applicable | | |
|  | D425* | Non-metastatic primary tumour of 5yo male | 🗶 | Not applicable | | |
|  | D458* | Matched metastatic recurrence of D425 | ✓ | 200 | NS medium  + 0.24% methyl cellulose  + centrifugation | 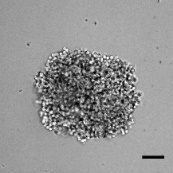 |
| **Group 4** | CHLA-01-MED^◊^ | Primary tumour with leptomeningeal dissemination of 8yo male | 🗶 | Not applicable | | |
|  | CHLA-01R-MED^◊^ | Matched metastatic recurrence of CHLA-01-MED | 🗶 | Not applicable | | |

*^◊^ Cell lines with matched primary and metastatic pairs. NS: neurosphere, yo: years old.

**Supplementary Table S4. Coefficient of variation analysis for SHH medulloblastoma 3D spheroids.** Spheroid diameter variation was calculated using coefficient of variation (CV) analysis for DAOY, ONS76, and UW228-3 spheroids on day 4 over three independent experimental repeats (n=1, 2, and 3). Variation within each plate (intraplate CV) and across three plates (interplate CV) is shown. CV values <20% are considered acceptable (Sittampalam et al., 2004).

| **Cell line** | **Intraplate CV (%)** | | | **Interplate CV (%)** |
| --- | --- | --- | --- | --- |
|  | **n=1** | **n=2** | **n=3** |  |
| DAOY | 4.13 | 2.91 | 3.21 | 7.60 |
| ONS76 | 7.31 | 4.64 | 2.59 | 13.33 |
| UW228-3 | 1.88 | 1.28 | 1.70 | 5.67 |

**Supplementary Table S5. Expression of Hedgehog GLI pathway markers in SHH medulloblastoma 3D spheroids.** FPKM values (number of fragments per kilobase of transcript per million mapped fragments) for DAOY and ONS76 3D spheroids for the gene set “PID_HEDGEHOG_GLI_PATHWAY”.

| **Gene** | **FPKM value** | |
| --- | --- | --- |
|  | **DAOY** | **ONS76** |
| AKT1 | 204.123 | 209.994 |
| ARRB2 | 23.7375 | 28.9029 |
| CREBBP | 22.8942 | 30.2913 |
| CSNK1A1 | 336.981 | 340.305 |
| CSNK1D | 0.998986 | 0.581985 |
| CSNK1E | 92.8961 | 60.8509 |
| CSNK1G1 | 61.8219 | 67.1765 |
| CSNK1G2 | 183.337 | 157.225 |
| CSNK1G3 | 23.0668 | 26.851 |
| FBXW11 | 38.0092 | 41.8712 |
| FOXA2 | 2.38045 | 0.587695 |
| GLI1 | 3.6073 | 4.98744 |
| GLI2 | 4.59576 | 5.45508 |
| GLI3 | 33.8093 | 31.9456 |
| GNAI1 | 19.2993 | 19.0437 |
| GNAI2 | 222.361 | 256.687 |
| GNAI3 | 152.076 | 196.053 |
| GNAO1 | 12.3272 | 9.43695 |
| GNAZ | 1.59005 | 1.66635 |
| GNB1 | 432.914 | 392.042 |
| GNG2 | 105.833 | 69.8053 |
| GSK3B | 52.2155 | 46.6218 |
| HDAC1 | 122.6 | 86.4663 |
| HDAC2 | 147.144 | 103.442 |
| IFT172 | 13.8682 | 12.8827 |
| IFT88 | 5.09549 | 4.6355 |
| KIF3A | 18.7583 | 24.5825 |
| LGALS3 | 174.633 | 204.293 |
| MAP2K1 | 26.1866 | 26.4367 |
| MTSS1 | 16.7374 | 16.0712 |
| PIAS1 | 17.2775 | 17.0588 |
| PRKACA | 101.787 | 104.468 |
| PRKCD | 6.6719 | 8.35563 |
| PTCH1 | 17.4839 | 17.5394 |
| RAB23 | 11.3235 | 12.6481 |
| RBBP4 | 216.076 | 205.516 |
| RBBP7 | 164.169 | 174.788 |
| SAP18 | 192.822 | 187.269 |
| SAP30 | 23.6306 | 21.1456 |
| SHH | 0.502638 | 0.064991 |
| SIN3A | 24.4199 | 26.6296 |
| SIN3B | 36.9545 | 31.564 |
| SMO | 35.5975 | 39.1598 |
| SPOP | 53.4926 | 58.9494 |
| SSPOP | - | 36.5064 |
| STK36 | 39.16 | 36.5064 |
| SUFU | 6.71804 | 6.90697 |
| XPO1 | 141.055 | 155.951 |

**Supplementary Table S6. Comparison of volume and luminescence as methods of analysing 3D spheroid drug response.** DAOY, ONS76, and UW228-3 spheroids were treated with increasing concentrations of etoposide for 72 hours. Non-linear regression analyses compared the fit of the drug response curve (hill slope) and IC_50_ concentrations generated by both spheroid imaging analysis (volume) and the CellTiter-Glo 3D cell viability assay (luminescence). There was no significant difference in either method of analysis for the two comparisons made.

| **Cell line** | **Method of analysis** | **Comparison 1: curve** | | **Comparison 2: IC_50_** | |
| --- | --- | --- | --- | --- | --- |
|  |  | **Hill slope** | **Significantly different?** | **IC_50_ concentration** | **Significantly different?** |
| DAOY | Volume | 1.879 | No  (p=0.8057) | 0.541 µM | No  (p=0.9811) |
|  | Luminescence | 1.500 |  | 0.539 µM |  |
| ONS76 | Volume | 1.841 | No  (p=0.8698) | 0.483 µM | No  (p=0.8075) |
|  | Luminescence | 1.560 |  | 0.472 µM |  |
| UW228-3 | Volume | 1.865 | No  (p=0.8482) | 0.870 µM | No  (p=0.3304) |
|  | Luminescence | 1.581 |  | 0.692 µM |  |

**Supplementary Table S7. Comparison of drug response in 2D monolayer versus 3D spheroid culture for SHH medulloblastoma cell lines treated with standard-of-care chemotherapeutics.** DAOY, ONS76, and UW228-3 cell lines were treated with increasing concentrations of etoposide, vincristine, cisplatin or lomustine in both 2D monolayer and 3D spheroid culture for 72 hours. CellTiter-Glo 3D cell viability assay was performed to compare the responses and viability was calculated as a percentage of the vehicle-treated controls. Non-linear regression analysis was used to compare the response of cell lines to each drug in both conditions and IC_50_ values (mean ± SEM) were calculated. Significant differences in IC_50_ concentrations and fold changes from 2D monolayer to 3D spheroid culture are shown. Measurable concentrations of drugs in patients is also displayed.

| **Drug** | **Measurable concentration in patients** | **Cell line** | **IC_50_ concentrations** | | **Significant difference in IC_50_ concentrations?** | **IC_50_ fold change**  (2D 🡪 3D) |
| --- | --- | --- | --- | --- | --- | --- |
|  |  |  | **2D monolayers** | **3D spheroids** |  |  |
| Etoposide (µM) | CSF: 0.21-0.3 µg/mL  Plasma: 7.01-10.47 µg/mL^8^ | DAOY | 0.06 ± 0.005 | 0.48 ± 0.042 | Yes (p≤0.0001) | x7 |
|  |  | ONS76 | 0.03 ± 0.001 | 0.39 ± 0.032 | Yes (p≤0.0001) | x12 |
|  |  | UW228-3 | 0.24 ± 0.015 | 0.66 ± 0.104 | Yes (p≤0.0001) | x1.75 |
| Vincristine (nM) | CSF: 0.76-0.95 nM  Serum: 14-27 nM^9^ | DAOY | 1.47 ± 0.06 | 3.94 ± 0.50 | Yes (p≤0.0001) | x1.68 |
|  |  | ONS76 | 2.30 ± 0.24 | 6.63 ± 0.79 | Yes (p≤0.0001) | x1.88 |
|  |  | UW228-3 | 0.90 ± 0.05 | 51.65 ± 30.95 | Yes (p≤0.0001) | x56.39 |
| Cisplatin (µM) | Plasma: 4-6 µg/mL^10^ | DAOY | 0.08 ± 0.005 | 0.91 ± 0.071 | Yes (p≤0.0001) | x10.38 |
|  |  | ONS76 | 0.05 ± 0.003 | 1.26 ± 0.101 | Yes (p≤0.0001) | x24.2 |
|  |  | UW228-3 | 0.59 ± 0.035 | 1.21 ± 0.086 | Yes (p≤0.0001) | x1.05 |
| Lomustine (µM) | Plasma: 0.8-0.9 µg/mL^11^ | DAOY | 35.80 ± 3.69 | 86.85 ± 8.91 | Yes (p≤0.0001) | x1.43 |
|  |  | ONS76 | 31.13 ± 4.01 | 81.97 ± 9.06 | Yes (p≤0.0001) | x1.63 |
|  |  | UW228-3 | 80.28 ± 7.69 | 87.39 ± 11.02 | No (p=0.6027) | x0.09 |

**Supplementary Table S8. Expression of genes associated with neuronal stem cell population maintenance in the spheroid migration model.** The raw data for the expression of genes from the gene set “GO_NEURONAL_STEM_CELL_POPULATION_MAINTENANCE” (GO:0097150) is displayed. Patient overall survival analysis was performed using the Cavalli medulloblastoma dataset on the R2 Genomics Analysis and Visualization Platform.

| **Gene** | **DAOY** | | | | | | **ONS76** | | | | | | **Patients**  **Bonferroni p-value** |
| --- | --- | --- | --- | --- | --- | --- | --- | --- | --- | --- | --- | --- | --- |
|  | **Spheroid** | **Migrating spheroid** | **Log2 fold change** | **p-value** | **FDR q-value** | **Significant?** | **Spheroid** | **Migrating spheroid** | **Log2 fold change** | **p-value** | **FDR q-value** | **Significant?** |  |
| ASPM | 10.8004 | 1.83792 | -2.55493 | 5.00E-05 | 0.00042 | yes | 10.4394 | 1.01084 | -3.36842 | 5.00E-05 | 0.00017 | yes | 1 |
| CDH2 | 68.271 | 17.0534 | -2.00121 | 5.00E-05 | 0.00042 | yes | 97.4611 | 12.4845 | -2.96468 | 5.00E-05 | 0.00017 | yes | 1 |
| DLL1 | 5.1918 | 16.5831 | 1.67541 | 0.0491 | 0.121665 | no | 2.75832 | 18.3096 | 2.73074 | 5.00E-05 | 0.00017 | yes | 3.60E-17^◊^ |
| FANCC | 14.8886 | 4.2707 | -1.80167 | 5.00E-05 | 0.00042 | yes | 13.3972 | 5.52886 | -1.27688 | 5.00E-05 | 0.00017 | yes | 0.127 |
| FANCD2 | 31.9082 | 11.1295 | -1.51954 | 0.04895 | 0.12139 | no | 37.4356 | 3.05505 | -3.61514 | 5.00E-05 | 0.00017 | yes | 1 |
| FOXO1 | 7.32084 | 9.57835 | 0.387769 | 0.471 | 0.573022 | no | 7.45639 | 11.3297 | 0.60356 | 0.21155 | 0.304096 | no | 0.121 |
| FOXO3 | 8.57444 | 8.96246 | 0.063851 | 0.85255 | 0.889334 | no | 8.50742 | 7.35893 | -0.20923 | 0.1369 | 0.21446 | no | 0.372 |
| FUT10 | 26.4848 | 13.0127 | -1.02525 | 0.05785 | 0.138384 | no | 29.6803 | 9.93103 | -1.57949 | 5.00E-05 | 0.00017 | yes | 1 |
| HES1 | 41.3428 | 54.8803 | 0.408652 | 0.2137 | 0.334524 | no | 34.7652 | 45.6218 | 0.392078 | 0.0029 | 0.007563 | yes | 0.151 |
| HES5 | 0.397737 | 36.9869 | 6.53905 | 5.00E-05 | 0.00042 | yes | 0.185416 | 11.1273 | 5.9072 | 0.00385 | 0.009733 | yes | 1.00E-05^◊^ |
| HOOK3 | 68.1371 | 56.9626 | -0.25843 | 0.5828 | 0.670202 | no | 71.4893 | 57.2814 | -0.31966 | 0.1439 | 0.22368 | no | 1.00E-03* |
| IGF2BP1 | 67.1131 | 0.308601 | -7.76471 | 5.00E-05 | 0.00042 | yes | 49.1852 | 0.229306 | -7.74481 | 0.0002 | 0.000641 | yes | 0.113 |
| JAG1 | 30.0781 | 74.2505 | 1.30369 | 0.0001 | 0.000779 | yes | 15.3234 | 50.108 | 1.7093 | 5.00E-05 | 0.00017 | yes | 5.90E-03^◊^ |
| MCPH1 | 5.98972 | 7.34934 | 0.295126 | 0.58395 | 0.671344 | no | 5.2379 | 5.30017 | 0.017049 | 0.93665 | 0.944492 | no | 0.105 |
| MMP24 | 0.282342 | 0.204575 | -0.46482 | 1 | 1 | no | 0.214303 | 0.143825 | -0.57534 | 1 | 1 | no | 1 |
| NOTCH1 | 11.2377 | 158.363 | 3.81682 | 5.00E-05 | 0.00042 | yes | 8.59075 | 143.912 | 4.06626 | 5.00E-05 | 0.00017 | yes | 1 |
| PCM1 | 58.8551 | 112.059 | 0.929025 | 0.0484 | 0.120411 | no | 54.5778 | 107.725 | 0.98096 | 0.00015 | 0.000488 | yes | 1 |
| PROX1 | 7.24728 | 2.57384 | -1.49352 | 0.00335 | 0.014737 | yes | 3.25671 | 2.04938 | -0.66822 | 0.0734 | 0.128259 | no | 1 |
| PRRX1 | 25.8806 | 35.5996 | 0.459988 | 0.3514 | 0.46075 | no | 22.4933 | 35.2623 | 0.64863 | 0.0246 | 0.049939 | yes | 0.623 |
| REST | 8.4675 | 6.86797 | -0.30205 | 0.48645 | 0.586655 | no | 9.24858 | 4.17996 | -1.14574 | 0.0017 | 0.004662 | yes | 0.06 |
| SOX2 | 16.1804 | 115.3 | 2.83308 | 5.00E-05 | 0.00042 | yes | 12.3499 | 81.5719 | 2.72357 | 5.00E-05 | 0.00017 | yes | 0.498 |
| SRRT | 409.526 | 964.246 | 1.23545 | 0.0044 | 0.018533 | yes | 370.462 | 647.096 | 0.804654 | 0.0011 | 0.003141 | yes | 1 |
| SS18 | 132.319 | 30.0611 | -2.13804 | 0.00465 | 0.019378 | yes | 122.55 | 43.7675 | -1.48543 | 0.00115 | 0.003272 | yes | 1 |

*low expression = poorer outcome; ^◊^ high expression = poorer outcome

**Supplementary Methods. Next-generation sequencing (NGS) of SHH medulloblastoma 3D spheroid models.**

**Sample preparation for NGS**

In order to perform NGS of SHH medulloblastoma metastasis models, it was essential to extract high quality RNA for library preparation and sequencing. However, RNA isolation from hydrogels proved particularly difficult due to the low yields of RNA obtained. To efficiently utilise our samples, we chose to adopt two NGS technologies: traditional mRNA sequencing, which required at least 100 ng of RNA per sample, and 3' UPX sequencing, a method used for low input samples that sequenced the 3' end of RNA near the poly-A tail.

Firstly, RNA was extracted from samples using the NucleoSpin RNA Plus kit (Macherey-Nagel). Following extraction, samples were shipped to QIAGEN Genomic Services (Hilden, Germany) where the subsequent steps of NGS were performed. Quality control of the RNA was performed using the Qubit RNA high sensitivity assay (Invitrogen) which quantified the amount of RNA in each sample.

Samples with 100 ng or more of RNA in total were suitable for mRNA NGS. Samples with lower RNA yields were lyophilised and resuspended in a smaller volume of RNase-free water. Another quality control step was performed to quantify the RNA. Samples with an RNA concentration above 1.4 ng/mL were appropriate for 3' UPX NGS. However, samples with a lower RNA concentration were unsuitable for either method of NGS.

Following RNA extraction and quantification, libraries were prepared and sequenced. For mRNA NGS, library preparation was carried out using the TruSeq Stranded mRNA library preparation kit. All prepared libraries successfully passed QIAGEN’s internal quality control checks and were subsequently sequenced on a NextSeq 500 Illumina sequencer. Following sequencing, quality control of the sequencing data was performed using FastQC analysis. All samples had high quality scores, indicating good technical performance of the sequencing. For 3' UPX NGS, library preparation was performed using the QIAseq UPX 3' Transcriptome kit. All prepared libraries successfully passed QIAGEN’s internal quality control checks and were sequenced on a NextSeq 500 Illumina sequencer. Following sequencing, FastQC analysis of the sequencing data was performed and all samples were considered suitable for downstream primary and secondary data analysis.

**Comparison of NGS techniques**

To compare the results of mRNA and 3' UPX analyses, we assessed five samples that were sequenced by both techniques. Firstly, genes were filtered based on FPKM (mRNA) and UMI (3' UPX) values of ≥10 and the number of genes identified for each technique were compared. mRNA sequencing identified around 30-50% more genes than 3' UPX sequencing (**Supplementary Figure S5**). This indicated that, as expected, the NGS techniques have different gene quantity outputs and, therefore, analysis based on individual gene levels would be unsuitable. To further compare mRNA and 3' UPX results, we then compared the differential gene expression analysis results between two sample sets (genes upregulated in spheroids on a HA matrix compared to spheroids on no matrix) to gain a broader overview of the NGS outputs. To do this, significantly differentially expressed genes were filtered based on their fold change (Log2 ≥2) and FPKM or UMI value (≥20). This generated a list of significantly differentially regulated genes between the two comparison groups. We compared the top 20 gene sets that overlapped with the Gene Ontology (GO) biological process gene set for each NGS method using Gene Set Enrichment Analysis (GSEA). Comparison of the gene set lists for mRNA and 3' UPX analysis showed that 75% of gene sets were shared between the two techniques (**Supplementary Table S4**). This indicates that although the results should not be compared on an individual gene level, a broader gene set level could be considered as a relevant and suitable comparison.

**Comparison of spheroid-matrix interaction**

Differential gene expression analysis was conducted to identify significantly up- and down-regulated genes involved in the spheroid-matrix interaction. This involved comparing two groups of samples: (1) “spheroid no matrix” and (2) “spheroid HA matrix”.

Analysis was firstly conducted within cell lines. For DAOY samples, for which only mRNA NGS was performed, significantly differentially expressed genes were filtered based on fold change (Log2 ≥1) and FPKM value (≥20). For ONS76 samples, the analysis was more complex as both mRNA and 3' UPX NGS was performed on samples in this comparison. ONS76 samples sequenced using mRNA NGS were filtered using the same method described for the DAOY samples. For 3' UPX differential gene expression analysis, significantly differentially expressed genes were filtered based on the same fold change threshold (Log2 ≥1) and mean UMI counts of ≥20. This resulted in two lists of differentially regulated genes for ONS76 samples generated for each NGS method. These lists were then compared and only the overlapping genes (i.e. the differentially regulated genes that were shared between the two NGS techniques) were used for further analysis.

The lists of differentially regulated genes for each cell line were then compared with each other. The overlapping genes which were shared between cell lines were further analysed using GSEA and the GO biological process gene set. Significance in overlap with the GO gene set was determined using an uncorrected p-value and a q-value (a p-value adjusted using the Benjamini-Hochberg False Discovery Rate (FDR) approach) to correct for multiple testing.


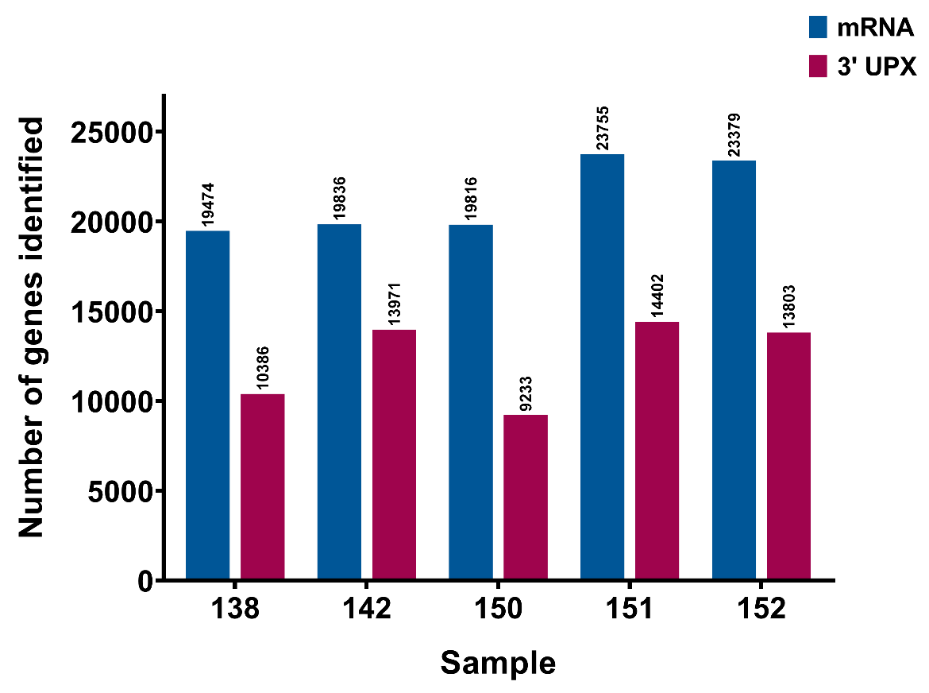


**Supplementary Figure S5. Number of genes identified in samples sequenced by both mRNA and 3’ UPX NGS methods.** Samples 138, 142, 150, 151, and 152 were sequenced by two next-generation sequencing (NGS) methods: mRNA and 3' UPX. Genes were filtered based on FPKM and UMI values of ≥10 and the number of genes identified for each technique were compared. mRNA sequencing identified around 30-50% more genes than 3' UPX sequencing.

**Supplementary Table S8. Comparison of top 20 gene sets upregulated in spheroids on a hyaluronan matrix identified by mRNA and 3’ UPX next-generation sequencing.** Differential gene expression analysis was conducted on samples sequenced by mRNA and 3' UPX NGS methods to generate lists of genes upregulated in spheroids on a HA matrix compared to spheroids on no matrix. Gene Set Enrichment Analysis was performed to identify the top 20 gene sets that overlapped with the Gene Ontology (GO) biological process database. The number of genes in each gene set (# genes) is shown. 75% of identified gene sets were shared by both mRNA and 3' UPX analyses (highlighted in blue).

| **mRNA** | | **3' UPX** | |
| --- | --- | --- | --- |
| **GO biological processes gene set** | **# genes** | **GO biological processes gene set** | **# genes** |
| Cell projection organisation (GO:0030030) | 148 | Cell projection organisation (GO:0030030) | 120 |
| Neurogenesis (GO:0022008) | 128 | Neurogenesis (GO:0022008) | 102 |
| Neuron differentiation (GO:0030182) | 113 | Central nervous system development (GO:0007417) | 76 |
| Cell projection assembly (GO:0030031) | 70 | Cilium movement (GO:0003341) | 25 |
| Neuron development (GO:0048666) | 95 | Neuron differentiation (GO:0030182) | 86 |
| Microtubule-based process (GO:0007017) | 77 | Cell projection assembly (GO:0030031) | 56 |
| Locomotion (GO:0040011) | 127 | Locomotion (GO:0040011) | 104 |
| Cilium organisation (GO:0044782) | 55 | Neuron development (GO:0048666) | 75 |
| Cytoskeleton organisation (GO:0007010) | 98 | Cellular component morphogenesis (GO:0032989) | 75 |
| Cellular component morphogenesis (GO:0032989) | 90 | Microtubule-based movement (GO:0007018) | 39 |
| Cell motility (GO:0048870) | 114 | Cell part morphogenesis (GO:0032990) | 57 |
| Central nervous system development (GO:0007417) | 80 | Biological adhesion (GO:0022610) | 83 |
| Cell morphogenesis involved in differentiation (GO:0000904) | 68 | Axoneme assembly (GO:0035082) | 22 |
| Cell part morphogenesis (GO:0032990) | 64 | Microtubule-based process (GO:0007017) | 59 |
| Regulation of cell differentiation (GO:0045595) | 111 | Cilium organisation (GO:0044782) | 43 |
| Cell morphogenesis involved in neuron differentiation (GO:0048667) | 58 | Cell morphogenesis involved in neuron differentiation (GO:0048667) | 51 |
| Positive regulation of developmental process (GO:0051094) | 93 | Microtubule bundle formation (GO:0001578) | 24 |
| Microtubule-based movement (GO:0007018) | 41 | Cytoskeletal organisation (GO:0007010) | 76 |
| Circulatory system development (GO:0072359) | 83 | Cell morphogenesis involved in differentiation (GO:0000904) | 56 |
| Cell-cell signalling (GO:0007267) | 100 | Ion transport (GO:0006811) | 87 |

1. Vinci, M. *et al.* Advances in establishment and analysis of three-dimensional tumor spheroid-based functional assays for target validation and drug evaluation. *BMC Biol.* **10**, 1–20 (2012).

2. Friedrich, J., Seidel, C., Ebner, R. & Kunz-Schughart, L. A. Spheroid-based drug screen: considerations and practical approach. *Nat. Protoc.* **4**, 309–324 (2009).

3. Vinci, M., Box, C., Zimmermann, M. & Eccles, S. A. Tumor spheroid-based migration assays for evaluation of therapeutic agents. in *Target Identification and Validation in Drug Discovery* **986**, 253–266 (Springer Protocols, 2013).

4. Kumar, K. S. *et al.* Computer-assisted quantification of motile and invasive capabilities of cancer cells. *Sci. Rep.* **5**, 1–13 (2015).

5. Chadwick, E. J. *et al.* A brain tumor/organotypic slice co-culture system for studying tumor microenvironment and targeted drug therapies. *J. Vis. Exp.* **2015**, 53304 (2015).

6. Meng, W., Garnett, M. C., Walker, D. A. & Parker, T. L. Penetration and intracellular uptake of poly(glycerol-adipate) nanoparticles into three-dimensional brain tumour cell culture models. *Exp. Biol. Med.* **241**, 466–477 (2016).

7. Neve, A., Kumar, K. S., Tripolitsioti, D., Grotzer, M. A. & Baumgartner, M. Investigation of brain tissue infiltration by medulloblastoma cells in an ex vivo model. *Sci. Rep.* **7**, 5297 (2017).

8. Kiya, K. *et al.* Penetration of etoposide into human malignant brain tumors after intravenous and oral administration. *Cancer Chemother. Pharmacol.* **29**, 339–342 (1992).

9. Jackson, D. V, Sethi, V. S., Spurr, C. L. & McWhorter, J. M. Pharmacokinetics of vincristine in the cerebrospinal fluid of humans. *Cancer Res.* **41**, 1466–1468 (1981).

10. Peng, B. *et al.* Cisplatin pharmacokinetics in children with cancer. *Eur. J. Cancer* **33**, 1823–1828 (1997).

11. Lee, F. Y. F., Workman, P., Roberts, J. T. & Bleehen, N. M. Clinical pharmacokinetics of oral CCNU (Lomustine). *Cancer Chemother. Pharmacol.* **14**, 125–131 (1985).
